# Expression analysis and in silico characterization of intronic long noncoding RNAs in renal cell carcinoma: emerging functional associations

**DOI:** 10.1186/1476-4598-12-140

**Published:** 2013-11-15

**Authors:** Angela A Fachel, Ana C Tahira, Santiago A Vilella-Arias, Vinicius Maracaja-Coutinho, Etel RP Gimba, Giselle M Vignal, Franz S Campos, Eduardo M Reis, Sergio Verjovski-Almeida

**Affiliations:** 1 Departamento de Bioquímica, Instituto de Química, Universidade de São Paulo, 05508-900 São Paulo, SP, Brazil; 2 Departamento RIR, Instituto de Humanidades e Saúde, Universidade Federal Fluminense, 28880-000, Rio das Ostras, RJ, Brazil; 3 Instituto Nacional de Câncer, 20231-050 Rio de Janeiro, RJ, Brazil; 4 Instituto Nacional de Ciência e Tecnologia em Oncogenômica, São Paulo, SP, Brazil

**Keywords:** Renal cell carcinoma (RCC), Unspliced intronic long noncoding RNAs, Antisense lncRNAs, Microarray analysis, Molecular markers, Gene expression correlation, Histone methylation, Histone acetylation, Evolutionary lncRNA conservation

## Abstract

**Background:**

Intronic and intergenic long noncoding RNAs (lncRNAs) are emerging gene expression regulators. The molecular pathogenesis of renal cell carcinoma (RCC) is still poorly understood, and in particular, limited studies are available for intronic lncRNAs expressed in RCC.

**Methods:**

Microarray experiments were performed with custom-designed arrays enriched with probes for lncRNAs mapping to intronic genomic regions. Samples from 18 primary RCC tumors and 11 nontumor adjacent matched tissues were analyzed. Meta-analyses were performed with microarray expression data from three additional human tissues (normal liver, prostate tumor and kidney nontumor samples), and with large-scale public data for epigenetic regulatory marks and for evolutionarily conserved sequences.

**Results:**

A signature of 29 intronic lncRNAs differentially expressed between RCC and nontumor samples was obtained (false discovery rate (FDR) <5%). A signature of 26 intronic lncRNAs significantly correlated with the RCC five-year patient survival outcome was identified (FDR <5%, p-value ≤0.01). We identified 4303 intronic antisense lncRNAs expressed in RCC, of which 22% were significantly (p <0.05) *cis* correlated with the expression of the mRNA in the same *locus* across RCC and three other human tissues. Gene Ontology (GO) analysis of those *loci* pointed to 'regulation of biological processes’ as the main enriched category. A module map analysis of the protein-coding genes significantly (p <0.05) *trans* correlated with the 20% most abundant lncRNAs, identified 51 enriched GO terms (p <0.05). We determined that 60% of the expressed lncRNAs are evolutionarily conserved. At the genomic *loci* containing the intronic RCC-expressed lncRNAs, a strong association (p <0.001) was found between their transcription start sites and genomic marks such as CpG islands, RNA Pol II binding and histones methylation and acetylation.

**Conclusion:**

Intronic antisense lncRNAs are widely expressed in RCC tumors. Some of them are significantly altered in RCC in comparison with nontumor samples. The majority of these lncRNAs is evolutionarily conserved and possibly modulated by epigenetic modifications. Our data suggest that these RCC lncRNAs may contribute to the complex network of regulatory RNAs playing a role in renal cell malignant transformation.

## Background

Renal cell carcinoma (RCC) is the most common cancer in adult kidney corresponding to nearly 3% of all adult malignancies worldwide [1], being an important cause of cancer morbidity and mortality [1]. Clear cell renal cell carcinoma (ccRCC) subtype is the most prevalent [2], making it especially important to identify the molecular changes associated with malignant transformation and with longer survival [3,4]. The malignant transformation has been associated to several changes in gene expression patterns, which are critical to several steps of tumor progression [5].

The noncoding RNAs (ncRNAs) exceed the number of protein-coding genes several fold [6], and both microRNAs (21–24 nt) (miRNAs) and long ncRNAs (≥ 200 nt) (lncRNAs) are now emerging as mammalian transcription key regulators in response to developmental or environmental signals [7-9]. The lncRNAs are classified based on intersection with protein-coding genes; when they map outside a protein-coding *locus* they are denominated long intergenic ncRNAs (lincRNAs) [9]. Otherwise they are classified as intronic, and in this case they can be either sense or antisense with respect to the direction of transcription of the host protein-coding gene in the *locus*[9].

Following the first reports of miRNA expression profiles associated with different types of cancer [10,11], several independent studies over the past five years identified a number of miRNAs differentially expressed in RCC that are correlated with malignancy [12-18] and with RCC subtypes classification [19,20]. In addition, a metastasis signature comprehending four miRNAs was recently described for ccRCC [21].

It has become evident that not only miRNAs but also lncRNAs are important players in cancer [22-27]. Studies on lncRNA expression have mainly been focused on the lincRNAs [28,29], essentially to simplify their analysis by avoiding possible complications arising from overlapping protein-coding genes [30]. Thus, recent transcriptome sequencing showed that lincRNAs are aberrantly expressed in a variety of human cancers [31]. A transcriptome sequencing study over a prostate cancer cohort identified the lincRNA *PCAT1* as implicated in malignancy progression [32]. In human lung adenocarcinoma, another lincRNA, *MALAT1,* has been associated with tumor metastasis [33] and is overexpressed in five other types of human cancers [34]. In a rare subtype of RCC, namely t(6;11) RCC, it has been described that *MALAT1* is fused to *TFEB* gene [35,36]. Recently, it has been shown that *Xist* lincRNA is a potent suppressor of hematologic cancer in mice [37].

Intronic lncRNAs constitute the major components of the mammalian ncRNA transcriptome [38], and the intronic lncRNAs are possibly related to a fine-tuning regulation of gene expression patterns across the entire genome [39]. Although thousands of putative intronic lncRNAs have been identified [9,38,40,41], it is yet to be determined which ones are functional. Also, it is a challenge to determine which ones are either independently transcribed or are by-products of pre-mRNA processing, with the levels of some of their intronic portions being independently regulated [38,42]. In fact, the mechanism of action of only a few intronic lncRNAs has been characterized in the context of cancer [42-44]. In addition, there is a number of studies reporting the correlation of expression patterns of intronic lncRNAs with cancer, such as intronic lncRNAs correlated to the degree of tumor differentiation in prostate cancer [45], intronic lncRNAs differentially expressed in primary and metastatic pancreatic cancer [46] and in dasatinib-treated chronic myeloid leukemia patients with resistance to imatinib [47]. In breast and ovarian cancer, Perez et al. [48] identified 15 aberrantly expressed ncRNAs, of which at least three are intronic [48]. In renal carcinoma, there are sparse studies regarding long noncoding RNAs. Our group previously identified seven intronic lncRNAs significantly deregulated in a set of six ccRCC tumor samples when compared with adjacent nontumor tissues [49]. Using a microarray approach, another study revealed tumor-associated lincRNAs when comparing gene expression profiles in six pairs of ccRCC and adjacent nontumor tissues [50].

In the present work, our study focused on the analysis of unspliced intronic lncRNAs, the class of lncRNAs that is the least studied one, in an attempt to point to possible new key molecules and pathways involved in renal carcinogenesis. In order to analyze gene expression patterns in tissue samples from RCC patients, we used herein two different microarray platforms enriched with probes for these intronic lncRNAs. We identified intronic lncRNAs whose differential expression was significantly correlated with RCC malignancy or with patient survival outcome. We also identified sets of intronic lncRNAs that are co-regulated in *cis* or in *trans* with protein-coding mRNAs encoding genes associated with transcriptional regulation and with kidney functions. Finally, our data demonstrate that RCC-expressed lncRNA *loci* are significantly associated with CpG islands and histone regulatory modifications typical of active RNA Pol II-transcribed genes, and that the intronic lncRNAs expression pattern in RCC is markedly tissue-specific and evolutionarily conserved.

## Results

### Expression signature of intronic long noncoding RNAs associated to malignancy in clear cell renal cell carcinoma

Based on our previous work with kidney tumor samples that identified a gene expression signature of 64 genes associated to ccRCC that included only 7 intronic lncRNAs [49], we looked for additional intronic lncRNAs differentially expressed between ccRCC and nontumor tissues. For this purpose, we analyzed eleven pairs of tumor (T) and matched adjacent nontumor renal tissue (N) samples from ccRCC patients. Clinical and pathological data of each patient are shown in Additional file 1: Table S1. Gene expression was measured with a non-strand-specific 4 k-element cDNA microarray platform that interrogates the expression of 722 intronic lncRNAs, 262 lincRNAs and 2371 protein-coding genes [45,49], now employing an improved T7 RNA-polymerase-based cRNA linear amplification and labeling protocol, as described under Methods.

**Figure 1:**
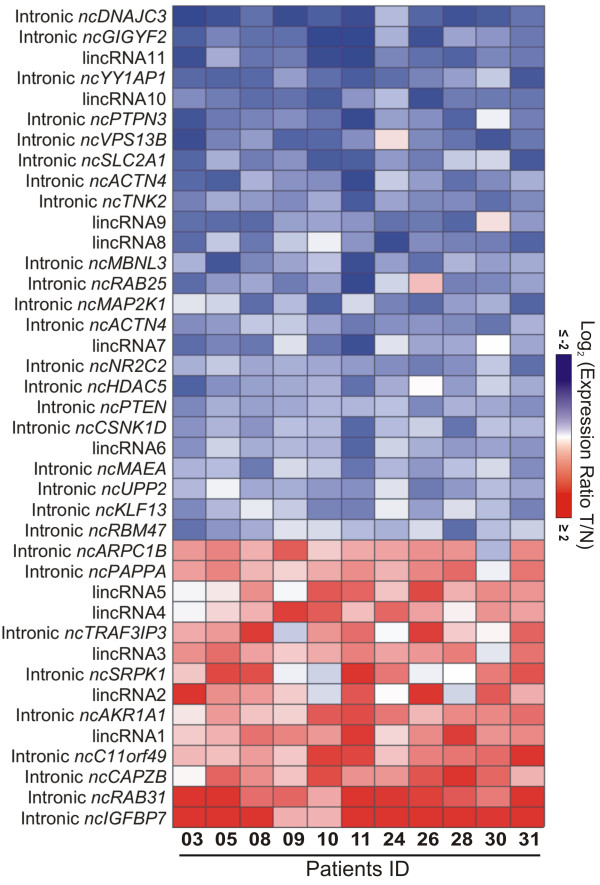
**lncRNA expression signature of malignancy in clear cell renal cell carcinoma (ccRCC).** Heat map of 40 differentially expressed lncRNAs (rows) identified in 11 ccRCC patients (columns). Patient ID numbers are shown at the bottom. (false-discovery-rate <5%; fold-change ≥1.5). There are 29 intronic lncRNAs (identified by their host-gene symbols) and 11 lincRNAs. Blue indicates lower expression, and red, higher expression in tumor (T) tissues in relation to adjacent nontumor (N) tissues.

A ccRCC-associated gene expression profile comprised of 29 intronic lncRNAs was identified with statistically significant differential expression, by comparing the expression of tumor and paired nontumor samples from eleven patients (FDR ≤5%, 1.5-fold change) (Figure 1). To minimize the contribution of each individual patient sample to the set of significantly altered genes [51] the statistical analysis has included a leave-one-out cross-validation procedure; essentially, one sample was removed at a time, and every time a new set of significantly altered genes was determined using the remaining ten samples, ultimately pointing to the most consistently altered gene set, which is common to all leave-one-out sets (see Material and methods for details). In addition, 9 non-annotated lincRNAs and 2 RefSeq lincRNAs were identified as significantly differentially expressed, totalizing 40 altered lncRNAs. Among the 40 lncRNAs, 26 were downregulated and 14 were upregulated in tumors when compared with nontumor tissues (Figure 1). The list of lncRNAs with altered expression is shown in Table 1.

**Table 1:** List of 40 lncRNAs differentially expressed in RCC from the present work: 29 intronic lncRNAs and 11 lincRNAs (FDR <5%; >1.5-fold change)

**GenBank accession of probe**^**§**^	**lncRNA type**	**RefSeq of host gene**	**Host gene symbol**	**Genomic coordinates**	**FDR (%)**	**Fold change of lncRNA**	**Average fold change of host gene**^**†**^
AW835362	Intronic	NM_001553	*IGFBP7*	chr4:57928550-57929060	0.00	4.88	-1.15 (2/4)
AW881130	Intronic	NM_006868	*RAB31*	chr18:9711672-9712160	0.00	3.72	**2.56 (5/5)**
BF881464	Intronic	NM_004930	*CAPZB*	chr1:19724054-19724494	0.00	2.03	1.34 (3/5)*
AW846722	Intronic	NM_024113	*C11orf49*	chr11:47169567-47169799	0.00	2.02	-0.34 (2/4)
AW815357	Intronic	NM_153326	*AKR1A1*	chr1:46029945-46030338	0.00	1.77	**-2.20 (3/3)**
AW937741	Intronic	NM_003137	*SRPK1*	chr6:35819568-35820194	2.46	1.7	-1.37 (3/4)
BF350736	Intronic	NM_025228	*TRAF3IP3*	chr1:209954933-209955401	1.93	1.65	1.39 (4/4)
CK327196	Intronic	NM_002581	*PAPPA*	chr9:119104917-119105402	1.86	1.63	**-3.49 (5/5)**
BF743551	Intronic	NM_005720	*ARPC1B*	chr7:98991157-98991537	1.37	1.6	**2.90 (5/5)**
AW748493	Intronic	NM_001098634	*RBM47*	chr4:40563872-40564416	3.16	-1.52	**-1.82 (4/4)**
CK327206	Intronic	NM_015995	*KLF13*	chr15:31628837-31629268	2.46	-1.53	1.19 (1/4)
CK327137	Intronic	NM_173355	*UPP2*	chr2:158886308-158886559	1.37	-1.55	**-6.05 (1/2)**
BE168993	Intronic	NM_005882	*MAEA*	chr4:1318234-1318651	1.68	-1.56	**-1.63 (2/4)**
BE181783	Intronic	NM_001893	*CSNK1D*	chr17:80226176-80226555	0.75	-1.63	-1.28 (3/3)*
AW836810	Intronic	NM_000314	*PTEN*	chr10:89630175-89630699	0.00	-1.63	**1.74 (4/4)**
BG010306	Intronic	NM_005474	*HDAC5*	chr17:42175003-42175469	0.75	-1.66	0.00 (0/3)
BF327015	Intronic	NM_003298	*NR2C2*	chr3:15052840-15053222	0.00	-1.7	-0.34 (0/5)
BF882783	Intronic	NM_004924	*ACTN4*	chr19:39203995-39204367	0.75	-1.74	-0.29 (1/5)
BF357721	Intronic	NM_002755	*MAP2K1*	chr15:66764897-66765436	0.00	-1.78	**1.52 (3/4)**
BF360792	Intronic	NM_020387	*RAB25*	chr1:156032114-156032418	0.75	-1.79	**-5.17 (4/4)**
CK327077	Intronic	NM_001170704	*MBNL3*	chrX:131621693-131622042	0.00	-1.9	-0.79 (0/5)
CK327106	Intronic	NM_005781	*TNK2*	chr3:195591793-195592189	0.00	-1.95	-1.16 (4/4)*
BF768459	Intronic	NM_004924	*ACTN4*	chr19:39200205-39200785	0.00	-2.01	-0.29 (1/5)*
BF368747	Intronic	NM_006516	*SLC2A1*	chr1:43409776-43410148	0.00	-2.01	**4.43 (5/5)**
BE080597	Intronic	NM_002829	*PTPN3*	chr9:112237298-112237614	0.00	-2.13	**-2.15 (5/5)**
BF332192	Intronic	NM_017890	*VPS13B*	chr8:100419550-100419768	0.00	-2.13	1.37 (3/5)
BE168995	Intronic	NM_018253	*YY1AP1*	chr1:155656314-155656660	0.00	-2.23	n.d.
CK327034	Intronic	NM_015575	*GIGYF2*	chr2:233592945-233593379	0.00	-2.45	1.16 (2/4)
BF368584	Intronic	NM_006260	*DNAJC3*	chr13:96432041-96432369	0.00	-2.81	**1.74 (1/1)**
BF368636	lincRNA1 RefSeq ncRNA	NR_028288	*TCL6*	chr14:96131134-96131552	0.00	1.92	-
AW880409	lincRNA2 RefSeq ncRNA	NR_003255 and NR_001564	*TSIX* and *XIST*	chrX:73042786-73043127	1.95	1.74	-
AW880864	lincRNA3	n.a.	n.a.	chr9:18430899-18431447	0.41	1.68	-
BF987841	lincRNA4	n.a.	n.a.	chr14:53107162-53107542	1.12	1.64	-
AW880828	lincRNA5	n.a.	n.a.	chr2:26955660-26956225	0.78	1.63	-
BF333219	lincRNA6	n.a.	n.a.	chr12:49324983-49325465	0.75	-1.63	-
BG009895	lincRNA7	n.a.	n.a.	chr21:19119867-19120311	0.00	-1.74	-
BE710971	lincRNA8	n.a.	n.a.	chr17:18176339-18176682	0.00	-1.93	-
BE718437	lincRNA9	n.a.	n.a.	chr17:56595754-56596085	0.00	-1.94	-
BF333731	lincRNA10	n.a.	n.a.	chr17:62118605-62119046	0.00	-2.19	-
AW996872	lincRNA11	n.a.	n.a.	chr15:58887770-58888280	0.00	-2.45	-

§ Probes are double-stranded cDNA, with the sequences that are indicated in the EST accession numbers from GenBank.

† Host protein-coding genes expression from a meta-analysis of ccRCC gene expression studies [54-58] obtained with the Oncomine™ Software tool: average fold change (FC) values in tumor relative to nontumor tissues of all studies are shown. The numbers in bold indicate FC values in the range -1.5 > FC > 1.5. The numbers in parenthesis represent: number of studies with significant differential expression of that gene/number of studies that have detected the expression of that gene.

* For this protein-coding gene more than one probe was present in some of the studies, and these additional probes showed an opposite expression pattern in the Oncomine Software. We show the most frequent pattern for this gene among all studies.

### Protein-coding genes differentially expressed in ccRCC and meta-analysis of malignancy related genes

To evaluate our microarray performance, we searched for protein-coding genes differentially expressed in our renal cancer samples and compared this set of genes with lists of protein-coding genes differentially expressed in ccRCC from nine published studies [5,49,52-58]. We identified a set of 217 protein-coding genes differentially expressed in our ccRCC samples relative to nontumor adjacent renal tissue (FDR <5%, leave-one-out cross-validation, 1.5-fold change) (Additional file 2: Figure S1; Additional file 3: Table S2). The meta-analysis is summarized in Table 2 and is described in detail in Additional file 3: Table S2. A total of 170 (78%) protein-coding genes are expressed in common between our study and the other nine previous studies. Most genes expressed in common (142/170; 83%) showed a concordant expression pattern.

**Table 2:** Summary of meta-analysis of the 217 protein-coding genes ccRCC signature from the present work with nine publicly available microarray studies comparing tumor and nontumor tissue samples from ccRCC patients

	**Takahashi et al. [**[52]**]**	**Skubitz et al. [**[5]**]**	**Higgins et al. [**[56]]*	**Lenburg et al. [**[54]]*	**Liou et al. [**[53]**]**	**Jones et al. [**[55]**]**	**Gumz et al. [**[57]]*	**Beroukhim et al. [**[58]**]**	**Brito et al. [**[49]**]**
# of genes in common	4	2	35	93	4	42	109	1	29
# of concordant genes^§^	4	2	29	78	4	38	94	1	29
**% of concordance**	**100.0**	**100.0**	**82.9**	**83.9**	**100.0**	**90.5**	**86.2**	**100.0**	**100.0**

§ Genes differentially expressed in the same direction (up or down) of that from our study.

* Lists of differentially expressed genes (p <0.05) obtained from Oncomine™ database. The lists of differentially expressed genes from the other studies in this Table were obtained directly from the published papers (see References).

Additionally, we looked at the expression of the 11 host protein-coding genes (among the 29) for which there were probes for the mRNA from the *loci* related to the intronic lncRNAs candidates. Of these, 10 protein-coding genes were detected as expressed in RCC (*IGFBP7*, *RAB31*, *PAPPA*, *ARPC1B*, *PTEN*, *HDAC5*, *NR2C2*, *MAP2K1*, *PTPN3*, *DNAJC3*). Only two were detected as significantly differentially expressed in RCC compared with nontumor, namely *RAB31* (fold-change =2.2) and *ARPC1B* (fold-change =1.84) (Additional file 3: Table S2). The fold-change and the direction of change of the protein-coding genes in tumors are in agreement with the literature data from the meta-analyses shown below.

Because of the limited representation of protein-coding genes in the 4 k array, we performed a meta-analysis with the ccRCC microarray studies in the literature [5,49,52-58], looking for protein-coding genes differentially expressed in the *loci* of the 29 intronic lncRNA candidates of our study. Of the 29 protein-coding genes, 28 were detected as expressed in at least one study included in our meta-analysis (Table 1, last column). Among them, we identified 13 genes with significantly altered expression in tumor compared to nontumor, displaying fold-changes greater than > |1.5|, of which 7 were altered in the same direction of the intronic lncRNA (concordantly changed) and 6 were altered in the opposite direction (inversely changed) (Table 1, last column).

### Intronic lncRNA expression profile is correlated to the patient survival outcome in RCC

Next, we asked if there was a signature of intronic lncRNAs associated to the patient survival outcome in ccRCC. We considered the lncRNA expression data of the paired and unpaired tumor samples from sixteen ccRCC patients who had a cancer-specific death or were disease-free within a 5-year follow-up after surgery (Additional file 1: Table S1). A supervised statistical analysis identified a 26-gene intronic lncRNA expression profile (Additional file 4: Figure S2; Additional file 5: Table S3) that was significantly correlated to the patient survival outcome (SAM statistical test, FDR ≤5%, combined with Golub’s discrimination score, p <0.01; see Material and methods for details). No lincRNAs in the array were identified as correlated to survival. Most of the altered intronic lncRNAs present in this signature (24/26, i.e. 92%) were down regulated in the disease-free group. Patient status (PS, Additional file 4: Figure S2B, first line) refers to the disease outcome within the 5-year follow-up after surgery, and it should be noted that it was the sole criterion used for the supervised statistical analysis of correlated lncRNA expression. For comparison, an additional eight clinical and pathological parameters related to each patient are shown (Additional file 4: Figure S2). Interestingly, a set of eight intronic lncRNAs was detected in common, both in the patient survival profile and in the ccRCC-associated gene expression profile described above (Additional file 5: Table S3, last column). Validation of the patient survival profile with an independent, larger patient cohort is warranted.

### Real-time qPCR validation assay

**Figure 2:**
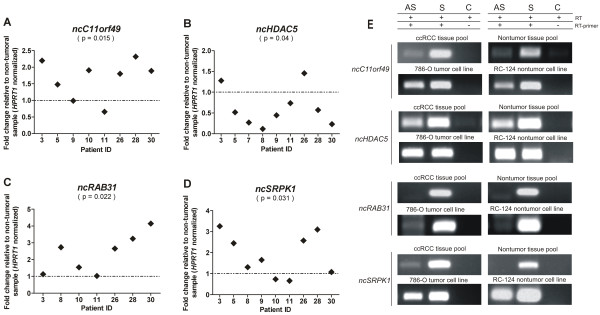
**Relative quantification and transcriptional orientation of intronic lncRNAs differentially expressed in ccRCC.** Expression of **(A)***ncC11orf49*, **(B)***ncHDAC5*, **(C)***ncRAB31* and **(D)***ncSRPK1* was evaluated in tumor and adjacent nontumor paired samples from clear cell RCC patients by qPCR. Tumor expression relative to paired nontumor in each patient sample is shown. lncRNA expression was normalized by *HPRT1* gene expression. The statistical significance of the differential expression was evaluated by the *t*-test (p < 0.05). **(E)** For each gene, strand-specific reverse transcription (RT) followed by PCR shows the presence of intronic messages transcribed from the antisense (AS) and/or the sense (S) strands, in a pool of 10 ccRCC tissues, in a pool of 10 matched nontumors, or in the 786-O tumor and the RC-124 nontumor kidney cell lines. A control **(C)** for the absence of RNA self-annealing during reverse transcription and for the absence of genomic contamination was performed with an RT reaction step without primer (+ RT, - RT primer), followed by PCR with the pair of primers for the corresponding lncRNA.

To further validate the microarray expression data, we performed independent measurements of RNA abundance in tumor samples using real-time quantitative PCR. The limited amount of RNA available from patient samples was a challenge, and we selected only eight intronic lncRNA candidates to perform these assays. In addition, due to the lack of available RNA from all patients, we could only test a fraction of the cohort. Four lncRNAs mapping to intronic regions, namely *ncC11orf49*, *ncHDAC5*, *ncRAB31* and *ncSRPK1*, showed a significant (p <0.05) differential expression between tumor and nontumor paired samples as measured by qPCR (Figure 2A-D); transcripts from these four *loci* showed an expression pattern comparable to the observed in the array, thus corroborating the differential expression observed in the microarray analysis (Pearson correlation r = 0.96). Real-time qPCR measurements for transcripts from other four intronic regions (*ncACTN4, ncIGFBP7*, *ncMAP2K1, ncPTEN*) presented high expression variability and could not be validated (data not shown).

### Transcriptional orientation assay

For the four intronic lncRNAs *ncC11orf49*, *ncHDAC5*, *ncRAB31* and *ncSRPK1* with differential expression in ccRCC validated by RT-qPCR assay, transcriptional orientation (sense and/or antisense) was measured by strand-specific reverse transcription followed by PCR (Figure 2E) in the ccRCC and nontumor patient tissues. Three *loci* showed evidence of both sense and antisense messages (*ncC11orf49, ncHDAC5* and *ncSRPK1*). For the *ncRAB31 locus*, only a transcript with the same (sense) orientation of the corresponding protein-coding mRNA was detected (Figure 2E). The pattern of strand-oriented expression detected in human kidney tissues (pool of ccRCC or nontumor samples) was reproduced in kidney human cell lines originated from tumor (786-O) and nontumor (RC-124) (Figure 2E). To ensure that the strand-oriented determinations were free from technical artifacts, a control for the absence of self-annealing during reverse transcription (RT) and for the absence of genomic contamination was performed with the RT reaction step without any primer, followed by PCR with the pair of primers for the respective lncRNA; no products were detected in the controls (Figure 2E, control lanes).

### ncHDAC5: characterization of the intronic lncRNA decreased in RCC

The intronic lncRNA *ncHDAC5*, which is expressed from the *locus* of the regulatory histone-modifying enzyme *HDAC5*, was chosen for a more detailed characterization, because we identified it as decreased in the malignancy profile and increased in the patients with a poor survival outcome. We extended the *ncHDAC5* transcript by 3′- and 5′-RACE-PCR with a fetal kidney RACE library, sequenced the products and determined the *ncHDAC5* RNA expressed in kidney to have 1695 nt (GenBank Accession JX899681). Stability of the *ncHDAC5* transcript was examined by the actinomycin-treatment assay, revealing a half-life of 42 min in the 786-O kidney tumor cell line (Additional file 6: Table S4A).

**Figure 3:**
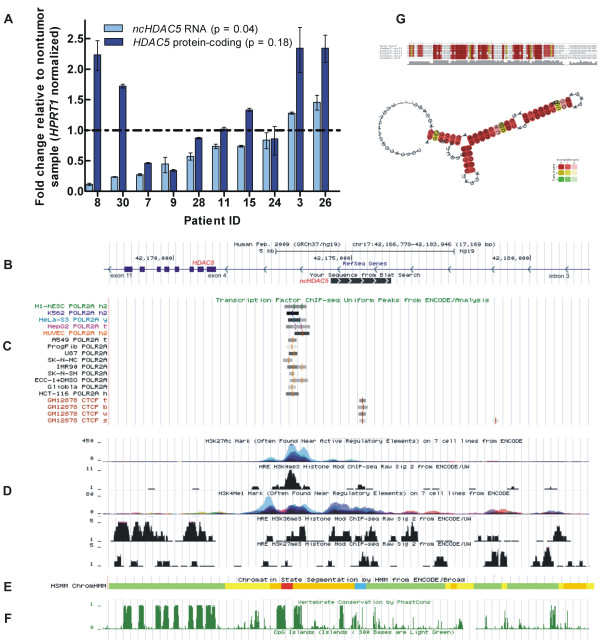
**Characterization of the intronic lncRNA expressed from the *HDAC5 locus*. (A)** Relative abundances of the *ncHDAC5* lncRNA (light blue) and of the *HDAC5* protein-coding mRNA (dark blue) are shown as fold change in the tumor relative to the matched nontumor sample for each of ten ccRCC patients. Patients are order according to the fold change of the *ncHDAC5*. **(B-F)** Regulatory and conserved elements from the ENCODE database are shown at the genomic region of the *HDAC5* protein-coding gene from intron 3 to intron 11. Arrowheads in **(B)** show the opposing directions of transcription of the *HDAC5* and the *ncHDAC5* RNAs. In **(C)** the RNA Polymerase II binding sites measured in 14 cell lines, and the CTCF transcriptional repressor insulator binding site are shown. In **(D)** the histone modification marks H3K27ac, H3K4me3, H3K4me1, H3K36me3 and H3K27me3 are shown. In **(E)** the HMM histone state segmentation annotation is shown, comprising a predicted active promoter (red), a strong enhancer (orange) and an insulator (blue) region. In **(F)** the vertebrate conservation and the CpG islands tracks are shown (no marks detected in the latter). **(G)** The most stable conserved secondary structure predicted by the RNAz tool (P = 0.99) for a segment within *ncHDAC5*. The segment spans 110 nt along the 1.7 kb-long lncRNA transcribed in the antisense direction in the *HDAC5 locus*.

The abundances of the *ncHDAC5* lncRNA and of the *HDAC5* protein-coding mRNA were measured in paired tumor and nontumor samples from ten ccRCC patients and are shown in Figure 3A. It is apparent that for the majority of patients (7/9) the expression level of the *ncHDAC5* was significantly lower (p <0.05) in tumor than in nontumor tissues (fold change relative to nontumor < 1) (Figure 3A, light blue). On the other hand, the protein-coding gene expression in tumor did not show a significant pattern of change relative to nontumor (p =0.18), the fold-change varying widely from 0.3 to 2.3 (Figure 3A, dark blue). In fact, these qPCR results confirm the 4 k-array expression measurements of *HDAC5* mRNA, which showed no significant changes in tumor compared with nontumor (see above). The expression of *HDAC5* mRNA was not correlated to the expression of *ncHDAC5* RNA (Pearson r =0.41, p =0.23), which indicates that the mRNA and the lncRNA are independently transcribed and/or independently regulated.

To further characterize the intronic *ncHDAC5* lncRNA, we looked at public genomic databases [59-61] for genomic marks of expression regulation in the genomic *locus* of *HDAC5* from intron 3 to intron 11 (Figure 3B-F). We identified RNA Pol II binding exclusively on intron 3, at the vicinity of exon 4, in 14 different cell lines (Figure 3C). Further downstream on intron 3, we found CTCF transcriptional repressor insulator binding (Figure 3C); it is known that insulators limit the activity of promoters and enhancers to certain functional domains. In addition, we identified the occurrence of the active enhancer-associated histone mark acetylation of histone 3 lysine 27 (H3K27ac) and of the promoter-associated histone mark trimethylation of histone 3 lysine 4 (H3K4me3) co-localized with the RNA Pol II binding site (Figure 3D). The regulatory-element-associated monomethylation of histone H3 lysine 4 (H3K4me1) as well as the active-transcription-associated histone mark trimethylation of histone 3 lysine 36 (H3K36me3) were identified along the genomic region encompassing the intronic *ncHDAC5* (Figure 3D). The repressive mark trimethylation of histone 3 lysine 27 (H3K27me3) was detected at low abundance in this *locus*, at a frequency similar to that of the exonic regions of the *HDAC5* gene, as expected for actively transcribed regions (Figure 3D). In fact, the HMM histone state segmentation analysis (Figure 3E) predicts an active promoter (red) at the left-hand part of the *ncHDAC5* locus, a strong enhancer region (orange) in the middle, and an insulator region (blue) at the right-hand side. Taken together, these ENCODE data suggest that the regulatory elements present in the *locus*, along with RNA Pol II can drive the transcription of *ncHDAC5* in the antisense direction, having the *ncHDAC5* TSS in the vicinity of the RNA Pol II binding site, as indicated in Figure 3B. It is likely that the sense transcript detected by strand-specific RT-qPCR in this intronic *locus*, reflects the presence of *HDAC5* pre-mRNA that may originate an independently regulated intron segment [38].

In addition, we determined that the genomic region upstream of the *ncHDAC5* putative TSS and within its transcription *locus* is evolutionarily conserved in vertebrates (Figure 3F). On the other hand, CpG islands were not detected upstream or within the *ncHDAC5* genomic region (Figure 3F). There was no evidence that *ncHDAC5* is a precursor of small RNAs, because no miRNA or snoRNA with sequence identity to the lncRNA were found in the public databases [62,63].

Finally, the *ncHDAC5* showed five distinct regions (ranging from 79 to 114 nt in length) where evolutionarily conserved secondary structures were predicted by RNAz tool (P > 0.5) (Additional file 6: Table S4B); the most significant secondary structure (P = 0.99) covering 110 nt is transcribed in the antisense direction, and its predicted folding is shown in Figure 3G.

### Functional associations of intronic antisense lncRNAs and protein-coding mRNAs in RCC

To extend the study of intronic antisense lncRNAs expressed in RCC we used a custom-designed 44 k oligoarray platform that allowed the detection of strand-specific expression in the intronic *loci*, by containing 10,525 single-stranded 60-mer oligonucleotide probes, essentially interrogating 15-fold more intronic *loci* than the array that we had used in the previous experiments. We focused on the antisense intronic lncRNAs, excluding the sense intronic ncRNAs from further analyses, because the antisense messages are admittedly transcribed independently from the protein-coding genes in the *loci*. The majority of RCC cases interrogated using this 44 k oligoarray were the clear cell subtype studied above (14 cases), and there were also papillary (2 cases) and chromophobe (1 case) subtypes; these seventeen tumor samples were randomly split into four pools, as indicated in Additional file 7: Table S5.

We identified 4303 antisense intronic lncRNAs as expressed in RCC from 3102 protein-coding gene *loci* (Additional file 8: Table S6). To verify their predicted noncoding status, we used the software Coding Potential Calculator (CPC) [64]. The CPC analysis pointed to a lack of protein coding potential of nearly all intronic antisense transcripts tested (4293/4303, 99.8%) (Additional file 8: Table S6). This finding indicates that the vast majority is indeed noncoding RNAs. To better describe our set of lncRNAs, it was cross-referenced with RefSeq annotations at the UCSC database (http://genome.ucsc.edu/). We found six RNAs (0.14%) already annotated as noncoding RNAs (Additional file 8: Table S6), indicating that the vast majority of our set are novel unannotated intronic antisense ncRNAs. To investigate if these are possible precursors of small RNAs, we cross-referenced the genomic coordinates of our 4303 antisense lncRNA set to snoRNA [62] and microRNA [63] datasets. Because microRNA precursor lengths are on average >1,000 nt, we extended the lncRNAs genomic coordinates by 1 kb at both the 3′- and 5′- ends. Only one ncRNA out of all 4303 ncRNAs mapped to a small RNA, namely U99 (Additional file 8: Table S6), suggesting that this set of antisense lncRNAs expressed in RCC has a diverse function other than being precursors of small RNAs.

**Figure 4:**
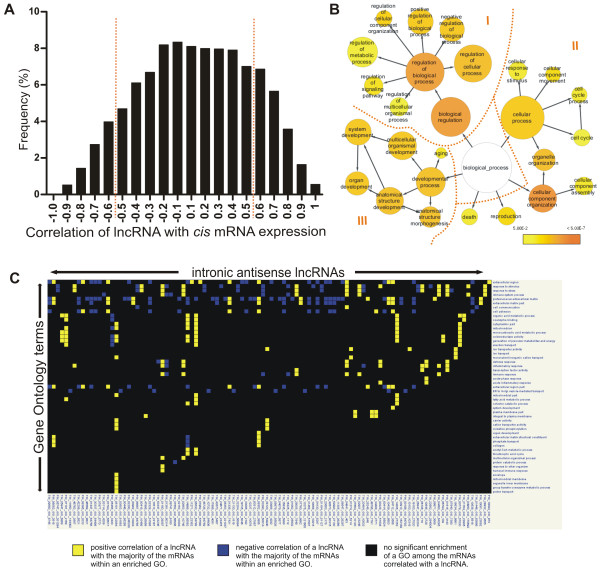
**Functional associations of intronic antisense lncRNAs expressed in RCC. (A)***Cis*-correlation analysis. Histogram of Spearman correlation values calculated using the expression levels of intronic lncRNAs and mRNAs expressed in 4303 gene *loci*, across RCC and three other human tissues (normal liver, prostate tumor and kidney nontumor). **(B)** GO enrichment analysis of the mRNAs correlated *in cis* with the lncRNAs from the same *loci* (Spearman correlation -0.5 > ρ >0.5; p <0.05; see red broken lines in panel **A**). Color scale indicates increasingly higher statistical significance of enriched GO terms: Yellow, p = 0.05; Dark orange, p <0.0001. **(C)***Trans*-correlation analysis. Module map of lncRNAs and GO enriched terms among *trans*-correlated mRNAs. Analysis was performed with the 20% most abundant lncRNAs (columns) that showed Spearman correlation values in the ranges -0.7 ≥ ρ ≥0.7 between its expression level in RCC and in three other human tissues (normal liver, prostate tumor and kidney nontumor) and the expression of mRNAs outside the host *locus* (correlation *in trans*; p <0.05); GO terms significantly enriched among *trans*-correlated mRNAs are shown in the rows (p <0.05 with Bonferroni correction). Colors indicate if the majority of the mRNAs within that GO is directly (yellow) or inversely (blue) correlated with the lncRNA. A black entry indicates no significant enrichment. The lists of GO enriched terms and of mRNAs belonging to each term for panels 4**B** and 4**C** are given in Additional file 10: Table S7.

Next, we investigated the patterns of co-expression of the antisense lncRNAs and the mRNAs expressed in *cis* (both expressed from the same *locus*) or in *trans* (expression of an antisense lncRNA correlated to the expression of mRNAs from other *loci*). We started with the 4303 intronic antisense lncRNAs expressed in renal cancer, and analyzed their expression pattern in RCC and in three human tissues previously studied by our group with the same microarray platform [40], namely normal liver, prostate tumor and kidney nontumor samples. For the *cis*-correlation analysis, Spearman correlation was calculated using the expression levels of each antisense lncRNA and the mRNA expressed from the same *locus* measured across RCC and the three tissue types. A total of 3467 (out of 4303; 81%) lncRNAs/mRNAs from the same *locus* were considered in the analysis because they were detected in all tissues. We identified a direct or inverse *cis*-correlation for the expression in the four tissues of 929 (929/4303 =22%) antisense lncRNA/mRNA from the same *locus* (Figure 4A and Additional file 8: Table S6). These lncRNAs/mRNAs had significant (p <0.05) correlation coefficients in the range -0.5 > ρ >0.5.

Next, we performed a gene enrichment analysis to identify Gene Ontology (GO) terms that were overrepresented among protein-coding genes whose expression was significantly *cis*-correlated to the expression of intronic antisense lncRNAs from the same *loci*. We found the term “biological regulation” as the most enriched general term (p < 5.00E-7) followed by “cellular component organization”, “cellular process”, “developmental process”, “reproduction” and “death” (Figure 4B). It is noteworthy that among all GO enrichment terms, the term “regulation” is present in 40% (61/152). Among the enriched “biological regulation” processes are the “regulation of cell growth”, “regulation of cell proliferation”, “regulation of cell communication”, the “positive regulation of protein metabolic process” and the “negative regulation of transcription from RNA pol II promoter” (Additional file 9: Figure S3).

Considering only the positive *cis*-correlation for GO enrichment analysis, 58 GO terms are enriched, and 98% (57/58) of those are present in the complete *cis*-correlation analysis. Regulation of cellular process is the most frequent GO term. Regulation is present in 34% (20/58) of all GO enriched terms. Considering only the negative *cis*-correlation for GO enrichment analysis, 60% (32/53) are related to regulation, being regulation of metabolic process the main enriched GO term. Of those, 32% (17/53) are exclusive GO terms that were not present in the complete *cis*-correlation analysis. All GO-enriched terms are shown in detail in Additional file 9: Figure S3 and listed in Additional file 10: Table S7.

We observed with the Spearman analysis described above that the expression of the majority of the antisense lncRNAs (78%) was not *cis*-correlated to the expression of the mRNA transcribed in the same *locus* (Figure 4A). Therefore, to investigate subsets of intronic antisense lncRNAs that were *trans*-correlated, we performed a Spearman correlation analysis comparing the level of each lncRNA with the expression levels of mRNAs from all genomic *loci* represented in the 44 k-array, again using the data from RCC and from the three other human tissues [40]. To favor the identification of biologically relevant regulatory RNAs, only the 20% most abundant intronic antisense lncRNAs in RCC (n = 860) were used for the *trans*-correlation analysis. A total of 693 antisense lncRNAs (out of 860; 81%) and 5438 mRNAs that were detected in all tissues were used to calculate a matrix of *trans* correlations. We identified inverse or direct high *trans*-correlation values (-0.7 ≥ ρ ≥0.7) between all 693 antisense lncRNAs and at least one of 5293 mRNAs from different genomic *loci* (out of 5438 mRNAs) (Additional file 11: Figure S4), which corresponds on average to the expression level of one antisense lncRNA being *trans*-correlated to the expression of 7.6 different expressed mRNAs in the four tissues studied.

Next, using Genomica software and the matrix of *trans* correlation as input, we constructed a module map of antisense lncRNAs versus GO enriched terms among the *trans*-correlated mRNAs (Figure 4C). We identified 106 intronic antisense lncRNAs positively and negatively associated to 51 enriched GO terms (p < 0.05, Bonferroni correction). Among those GOs with correlated lncRNAs are “response to stress”, “inflammatory response”, “metabolic process”, “immune response”, “RNA processing”, “response to stimulus”, and “ion transporter activity” (Figure 4C; Additional file 10: Table S7D).

### Intronic antisense lncRNAs expressed in RCC are enriched in genomic marks that suggest an independent gene expression regulation

To determine if regulatory elements occur at and are frequent in the genomic regions of the intronic antisense lncRNAs expressed in RCC, we compared the overlap distribution of genomic coordinates of these lncRNAs with datasets of genomic coordinates of Cap Analysis Gene Expression (CAGE) tags from PolyA+ RNA-derived libraries from 35 cell lines [65], of predicted CpGs islands [66], HMM active promoter prediction [59] and of ChIP-seq data for RNA Polymerase II binding site [65] and histone modification marks [59,60,67]. A random sequences set was used as control. Because the transcripts we had identified as expressed in RCC are mainly polyA+, given that our microarray experiments were performed using oligo-dT primed cDNA synthesis and labeling, we chose to use the PolyA+ RNA-derived ENCODE datasets.

**Figure 5:**
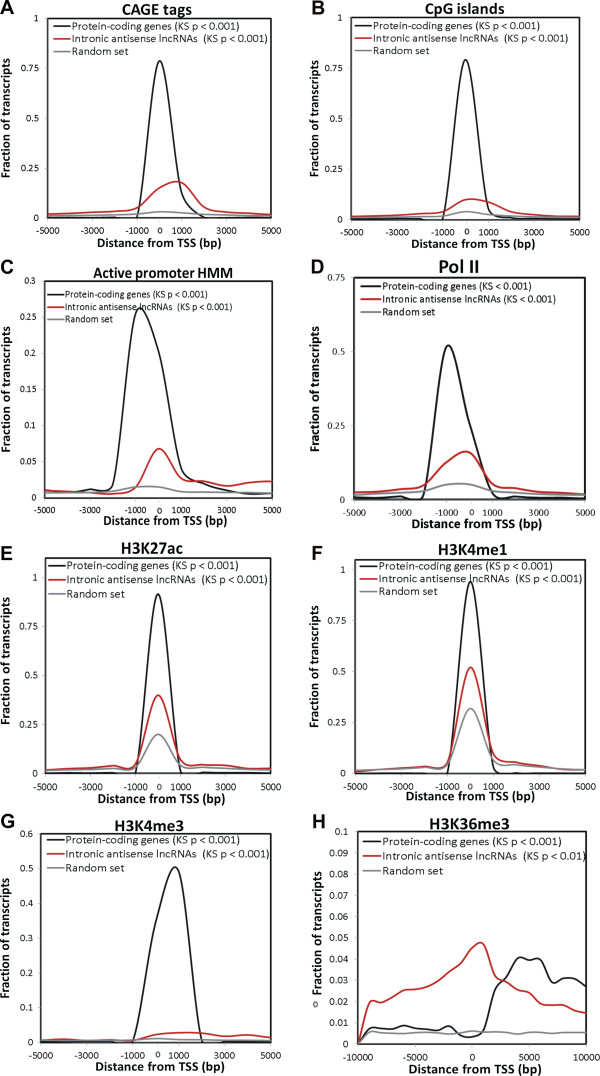
**Regulatory genomic marks associated with intronic antisense lncRNAs expressed in RCC.** Red lines show the abundance distribution of CAGE tags **(A)**, CpG islands **(B)** and histone marks **(C-G)** within a distance of 5 kb from the TSSs of the intronic antisense lncRNAs expressed in RCC. For comparison, abundance distribution of these marks for an equal number of protein-coding mRNAs (black lines), or for a control set of randomly selected intronic genomic sequences with the same length of the expressed lncRNAs (grey lines) were calculated. **(A)** CAGE tags, **(B)** CpG islands, **(C)** active promoter HMM predictions, **(D)** RNA polymerase II binding sites, **(E)** transcriptional activation histone mark H3K27ac, **(F)** transcriptional activation histone mark H3K4me1, **(G)** promoter-associated histone mark H3K4me3, and **(H)** activating-associated histone mark H3K36me3. In parentheses are the significance p-values of Kolmogorov-Smirnov (KS) statistical tests for differences in abundance distribution in relation to the control random set.

A significant association of CAGE tags with the putative antisense lncRNA TSSs was identified (Figure 5A). This finding is analogous to the reported presence of the 5′ cap modification at the TSS of lincRNAs [29]. CAGE tags are mostly present within the first kb from the known TSS of antisense lncRNAs and of mRNAs. This distribution is statistically different (Kolmogorov-Smirnov (KS) test p < 0.001) from that observed for the control random sequences set (Figure 5A). Next, we identified a significant association (KS test p < 0.001) between the predicted TSS of intronic antisense lncRNAs and CpG islands (Figure 5B), active promoter HMM predicted regions (Figure 5C) or RNA polymerase II binding site (Figure 5D).

We also identified a significant association between transcriptional activation histone marks H3K27ac (Figure 5E) or H3K4me1 (Figure 5F) and the putative TSSs of the intronic antisense lncRNAs (KS test p < 0.001); the analysis was performed with data from seven different human cell lines [59]. We looked at histone modification marks in renal tissue datasets [60], and found that the promoter-associated H3K4me3 mark (Figure 5G) and the activation-associated H3K36me3 mark (Figure 5H) showed a statistically significant higher frequency (KS test p < 0.001) at the genomic regions of the intronic antisense lncRNAs transcribed in RCC. The transcriptional repressive H3K27me3 mark was not identified in the renal tissue public data [60] at the TSSs of the antisense lncRNAs (data not shown); this was expected because these lncRNAs are the ones detected as expressed in RCC.

### Intronic antisense lncRNAs expressed in RCC are specifically expressed in other tissues

**Figure 6:**
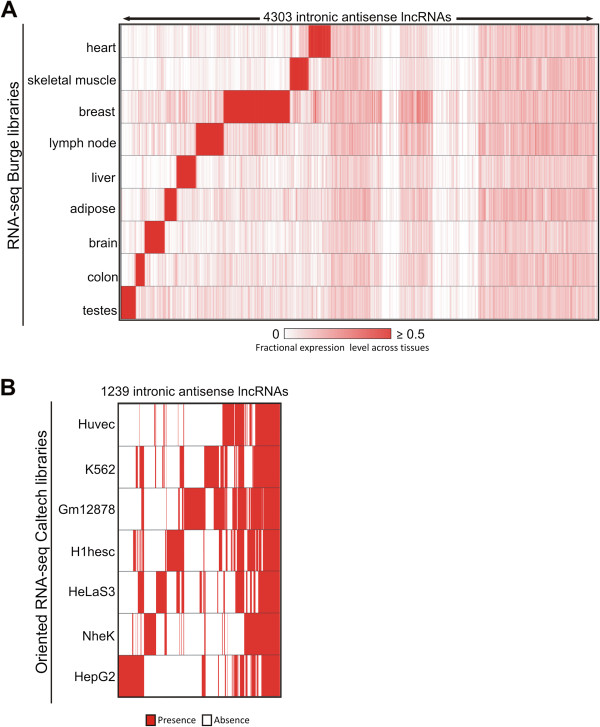
**Tissue expression pattern of intronic antisense lncRNAs. (A)** Heat map representing the abundance of 4303 RCC-expressed intronic antisense lncRNAs (columns) across other nine human tissues (rows) based on public RNA-seq data [68]. Color intensity represents fractional density expression of each lncRNA across all tissues (see Material and methods for details). There are 628 lncRNAs (out of the 4303; i.e. 15%) at the right hand side of this panel that were exclusively detected in RCC. **(B)** Heat map indicating the presence (red) or the absence (white) of 1239 RCC-expressed lncRNAs (columns) in seven human cell lineages (rows) from public strand-oriented RNA-Seq libraries [69]. These 1239 intronic antisense lncRNAs represent 29% of the 4303 lncRNAs detected in RCC; the other 3064 lncRNAs (71%) were detected exclusively in RCC, not in the cell lines (not shown). Expression data was hierarchically clustered.

To investigate the tissue-specificity of the 4303 intronic antisense lncRNAs expressed in RCC we cross-referenced the genomic coordinates of our dataset with the coordinates of RNA-seq reads from nine human tissues [68] (Figure 6A) and with RNA-seq data of strand-oriented libraries from seven human cell lines [69] (Figure 6B). In the human tissues analysis, we found that 15% of the antisense lncRNAs (628 out of 4303) were detected only in RCC (Figure 6A). A total of 3675 lncRNAs were detected in at least one of the nine tissues (Figure 6A). In the strand-oriented data from human cell lines, we found that 71% of the antisense lncRNAs (3064 out of 4303) were detected only in RCC (not shown). A total of 1239 lncRNAs (out of 4303, i.e. 29%) were detected in at least one out of the seven cell lines (Figure 6B). A similar well-marked expression pattern was observed for protein-coding genes across tissues and cell lines in RCC (Additional file 12: Figure S5), with the notable exception that the majority of these protein-coding genes (94%, i.e. 5296/5632) were detected in at least one of the strand-oriented RNA-seq data from the human cell lines.

### Intronic antisense lncRNAs expressed in RCC are evolutionarily conserved

**Figure 7:**
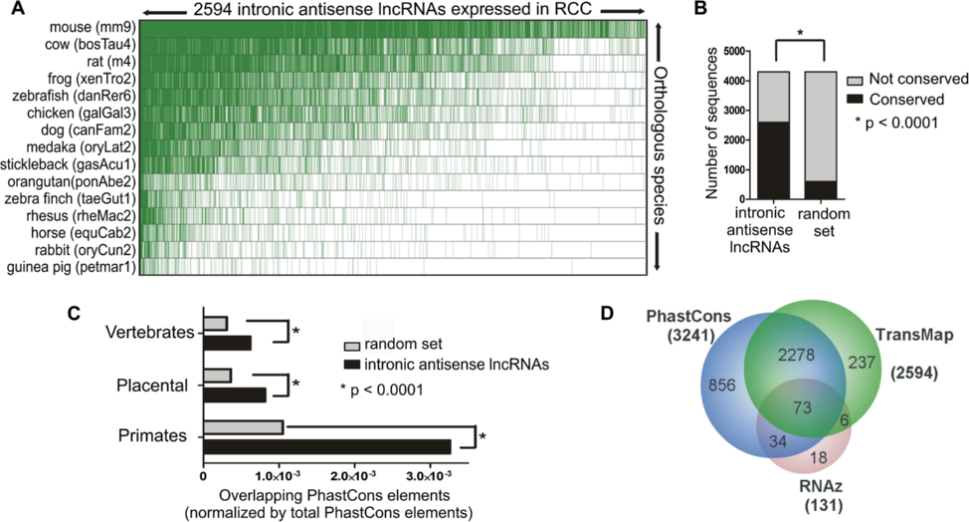
**Conservation analysis of intronic antisense lncRNAs expressed in RCC. (A)** TransMap cross-species cDNA alignments in 15 vertebrate species (rows; species common name and library version in brackets) of 2594 intronic antisense lncRNAs expressed in renal tissue and with conserved expression in at least one species (green dashes show expression conservation). **(B)** Bar graph of the TransMap analysis showing a higher proportion of expression conservation of the lncRNA dataset compared with a random sequence dataset (Fisher test p <0.0001). **(C)** DNA sequence conservation of antisense lncRNAs within vertebrates, placental and primates groups. Black bars show the number of lncRNAs, and gray bars the number of random genomic regions, overlapping PhastCons elements (see Material and methods for details). Asterisks show statistically significant differences in the number of overlapping elements (Fisher test p <0.0001). **(D)** Venn Diagram of three different conservation analyses of the intronic lncRNAs expressed in RCC: RNAz predicted secondary structure conservation for 131 lncRNAs, PhastCons DNA sequence conservation for 3241 lncRNAs and TransMap expression conservation for 2594 lncRNAs.

Expression conservation was evaluated by comparing the intronic antisense lncRNAs detected in RCC with cDNAs expressed in 15 vertebrate species that are compiled in the TransMap cross-species alignments [70]. This analysis revealed that 60% of the intronic antisense lncRNAs expressed in RCC (2594 out of 4303) are expressed in at least another species (Figure 7A). There is a higher proportion of expression conservation across the species in the lncRNA dataset compared with 10 control random sets of sequences extracted from the human genome (Fisher test p < 0.0001) (Figure 7B).

To further explore the conserved pattern of expression of these 4303 intronic antisense lncRNAs, we compared them with the 4858 introns harboring functional antisense ncRNAs recently identified by large scale RNA-seq in the mouse lung in response to inflammation [38]. A total of 1220 intronic regions could unequivocally be mapped to human genomic *loci*, and their corresponding coordinates were cross-referenced to the coordinates of the 4303 intronic antisense lncRNAs expressed in RCC. A total of 53 lncRNAs were detected as expressed in common both in mouse and in human, at syntenic *loci*, and the genomic coordinates are given in Additional file 8: Table S6. The length of overlap was in the range of 30 to 1228 bases among the 53 lncRNAs (Additional file 8: Table S6). We found a significantly higher proportion of expression overlap between mouse and RCC (53 out of 4303 intronic *loci* expressed in RCC) compared with a control random set of lncRNA sequences extracted from the subset of lncRNAs with no evidence of expression in RCC, among the entire set of intronic antisense lncRNAs probed in the 44 k array (overlap of 23 out of 4303 random intronic *loci* with no evidence of expression in RCC) (Fisher test p <0.001).

Comparison of the genomic coordinates of the 4303 intronic antisense lncRNAs expressed in RCC with those from conserved DNA elements identified in vertebrates, placental mammalians and primates (PhastCons, [61]) revealed a significant enrichment as compared with a random set of genomic sequences used as a control (Fisher test p < 0.0001) (Figure 7C). RNAz analysis [71] predicted secondary structure conservation for 131 intronic antisense lncRNAs (Additional file 8: Table S6 and secondary structures at http://verjo101.iq.usp.br/sites/projetosLab/fachel/structures/results.html). There are 73 antisense lncRNAs in common to all three conservation analyses described above (Figure 7D).

## Discussion

In the present study, we determined the expression pattern of a collection of intronic lncRNAs in clear cell RCC patients and identified candidates that might play a role in renal cancer biology. There are only two published studies of lncRNAs in RCC so far: our previous study [49] that identified for the first time seven intronic lncRNAs differentially expressed in RCC among a protein-coding gene signature; and the work of Yu *et al*. that identified 626 lncRNAs differentially expressed between tumor and nontumor tissue in 6 clear cell RCC patients. These authors used a microarray that essentially probed intergenic lncRNAs [50] and they validated by qPCR four transcripts, being three intergenic lncRNAs (ENST00000456816, X91348 and NR_024418); one was not a lncRNA, but rather the non-coding 3′-end portion of the *TMEM72* protein-coding gene (BC029135).

We identified 29 lncRNA transcripts originated from intronic regions and additionally 11 from intergenic regions, resulting in a ccRCC-associated gene expression profile comprised exclusively of lncRNAs. From this set, there are three intronic lncRNAs from the *ACTN4*, *HDAC5* and *SLC2A1 loci* identified as down-regulated both here and in our previous study [49] using the same microarray platform. This partial overlap (3 out of the 6 intronic lncRNAs described in Ref. [49]) is possibly related to the more stringent statistical criteria presently used, namely the leave-one-out approach that minimizes the contribution of each individual patient to the set of significantly altered genes when a small patient cohort is analyzed [51,72].

The comparison of our 217 protein-coding gene profile with nine published studies of differentially expressed protein-coding genes in ccRCC [5,49,52-58] verified that the vast majority (83%) of the genes in common (142/170) presented a concordant pattern of expression (Table 2), thus validating the present analysis as representative of the ccRCC biology.

Besides a set of intronic lncRNAs potentially involved in carcinogenesis, the present study identified a set of 26 intronic lncRNAs that were correlated to the survival of ccRCC patients. From this set, eight lncRNAs were identified as altered in both the malignancy and the survival outcome expression profiles, and they are transcribed from the *loci*: *ACTN4, CSNK1D, DNAJC3, GIGYF2, HDAC5, PTPN3, RAB25* and *VPS13B*. To the best of our knowledge, this is the first study suggesting lncRNAs as correlated to the patient survival outcome in RCC. Regarding other types of ncRNAs, there are at least two miRNA expression studies that had identified candidates correlated with patient survival outcome in RCC [21,73]. The lncRNAs identified in the present work may contribute to future studies focusing on lncRNAs as molecular markers in RCC oncology.

There are few examples of well-characterized lncRNAs associated with RCC. The lincRNA *GAS5* is a well described tumor suppressor in breast cancer [74], and very recently it was described in prostate cancer cell lines [75] and in RCC [76]. A decreased expression of the lincRNA *GAS5* is associated to RCC genesis and progression, and its overexpression is associated to cell proliferation inhibition and apoptosis induction [76]. Another example includes two antisense lncRNAs at the 5′ (5′aHIF-1α) and 3′ (3′aHIF-1α) ends of the human *HIF-1α* gene that are expressed in human kidney cancer tissues [77].

In cancer, there are a few examples of the mechanisms of action of intronic lncRNAs. Our group described the intronic antisense and unspliced lncRNA *ANRASSF1* that causes the epigenetic *in cis* downregulation of the tumor suppressor *RASSF1A* gene and increases cell proliferation [43], and its expression is higher in prostate and breast cancer cell lines compared with nontumor cells [43]. Guil *et al.*[42] identified that overexpression of the sense intronic lncRNA from the *SMYD3 locus* caused the epigenetic *in cis* regulation of *SMYD3* and a decrease in colorectal cancer cell line proliferation [42]. The androgen-regulated intronic antisense lncRNA *CTBP1-AS*[44] appears to be a key antisense ncRNA that acts as both *cis-* and *trans-*regulator of gene expression. The *CTBP1-AS* lncRNA promotes prostate cancer growth through sense-antisense repression of the transcriptional co-regulator *CTBP1* transcribed from the same *locus* (*cis-*regulation), and through a global epigenetic regulation of tumor suppressor genes (*trans-*regulation) [44]. In fact, the intronic and also the intergenic lncRNAs play important epigenetic roles in cancer [78].

We decided to study the intronic lncRNA *ncHDAC5* in more detail because it showed a decreased expression in ccRCC tumor compared with nontumor tissue that was confirmed by qPCR, and because its increased expression seems to be associated to the cancer-related death after surgery in RCC, as suggested by our patient survival outcome analysis. We determined that *ncHDAC5* is an unspliced long transcript (1.7 kb long), detected in the antisense and sense directions relative to the protein-coding gene histone deacetylase 5 (*HDAC5)*. It has a short half-life of 42 min compared with other well studied lncRNAs, such as *Air*, *Kcnq1ot1* and *Xist*, which have half-lives of 2.1, 3.4 and 4.6 h, respectively [79], with an evolutionarily conserved secondary structure. The absence of association between the expression of *ncHDAC5* and the protein-coding mRNA *HDAC5*, determined by qPCR and by a meta-analysis of five kidney cancer gene expression studies (Table 1), suggests a *locus* independent function, with the *ncHDAC5* possibly acting in *trans* to regulate protein-coding genes (see the discussion on *trans* regulation below). Unfortunately, a probe for this *ncHDAC5* was not present in the 44 k oligoarray that was used for assessing the *trans* correlation of expressed lncRNAs/mRNAs, and it was not possible to determine the *ncHDAC5* candidate target mRNAs by our co-expression analysis.

An in silico analysis indicated the presence of RNA Pol II binding and of the histone marks H3K27ac and H3K4me3 at ~1.5 kb upstream of the putative TSS of an antisense *ncHDAC5* transcript in the *HDAC5 locus*. Considering the lack of methylation marks in the vicinity of the lncRNA, this observation opens an interesting possibility of transcriptional regulation of the antisense lncRNA *ncHDAC5* by histone acetylation. It is in line with the result recently described for the lncRNA-LET, a lncRNA generally downregulated in carcinomas, that was shown to be repressed by histone deacetylase 3 under hypoxic conditions [80]. Interestingly, the transcriptional-activation-associated H3K4me1 and H3K27ac histone modification marks at human enhancers have been described as related to a cell-type specific protein-coding gene expression [81]. The TSSs at the lncRNA *ncHDAC5 locus* as well as at the *loci* of the other intronic antisense lncRNAs expressed in RCC were enriched with both histone marks, in agreement with the fact that the intronic lncRNAs tend to have a tissue-specific pattern of expression [9], thus supporting a possible cell-type specific modulation of intronic antisense lncRNAs by histone methylation and acetylation.

Because the intronic lncRNAs revealed a promising well-defined pattern of altered expression in RCC, and there is scarce data about this ncRNA class in RCC, we extended our study to the antisense intronic lncRNAs using a custom-designed strand-specific 44 k-element microarray that contained 15-fold more probes for lncRNAs than the 4 k-array that we had previously used. With this new platform, we identified 4303 antisense intronic lncRNAs expressed in RCC; we found that 4061 out of the 4303 antisense lncRNAs have not been previously reported in the Yu et al. study [50] as being expressed in RCC, which is in agreement with the fact that Yu et al. [50] used a microarray that probed mostly intergenic lncRNAs. In addition, only six lncRNAs are already annotated as RefSeq noncoding RNAs (Additional file 8: Table S6). In fact, the most recent catalog of human intronic lncRNAs comes from the GENCODE project [9], which documented the intronic lncRNAs expressed in 12 human normal tissues. Thus, the present study is a contribution towards the generation of a catalog of intronic antisense lncRNAs expressed in renal cancer.

The set of 4303 intronic antisense lncRNAs expressed in renal cancer identified in the present study probably has diverse functions, other than being precursors of small RNAs, because only one lncRNA mapped to a known small RNA sequence (U99, Additional file 8: Table S6). We found that 22% of the intronic antisense lncRNAs have expression levels in RCC, normal kidney, normal liver and tumor prostate that are correlated in *cis* to the expression levels of the mRNA from the same *locus*. These lncRNAs correlated in *cis* are transcribed from *loci* enriched with genes related to regulation, including the term “Regulation of Transcription from RNA polymerase II”, as seen when analyzing together the positively and negatively *cis*-correlated antisense lncRNA/mRNA as well as when analyzing only the positively *cis*-correlated transcripts (Additional file 9: Figure S3). Our group has described a similarly enriched GO term when analyzing the host gene *loci* of the 30% most abundant intronic antisense lncRNAs, without considering any expression correlation between the ncRNAs and the mRNAs [40]. Now we point to this GO term enrichment for those *loci* expressing the antisense lncRNAs and the mRNAs in a correlated manner, reinforcing the suggestion that the lncRNAs might *cis*-regulate the expression of the genes involved in “Regulation of Transcription” and/or that the antisense lncRNAs and the mRNAs might be controlled by a similar regulatory event in these *loci*.

We found that the expression of the majority of the intronic antisense lncRNAs was not correlated to the expression of the mRNA from the same *locus*, and those are most likely regulated in an independent way of the mRNAs. Among these, we identified a set of antisense lncRNAs whose expression in RCC, normal liver, prostate tumor and kidney nontumor tissues was positively or negatively correlated in *trans* to the expression levels of sets of mRNAs belonging to enriched GO terms such as “Inflammatory response” and “Response to stress”; these protein-coding genes may be related to the cellular renal cancer context, and the correlated lncRNAs are candidates to be acting in *trans* to regulate their expression. The present GO analyses support the proposal that ncRNAs might be part of a fine-tuning regulatory network in the cells [82-84].

Our computational analysis has generated a list of 4303 intronic antisense lncRNAs expressed in RCC (Additional file 8: Table S6) that includes subsets associated to CpG islands, CAGE tag marks, RNA pol II binding site, promoter-associated chromatin marks, tissue-specificity and evolutionary conservation. The set of 53 intronic antisense lncRNAs expressed in common at syntenic *loci* in human and mouse represent good candidates for subsequent in-depth biological follow up work; the low overlap may be related to the known tissue-specific expression of lncRNAs [8,41] and to the known tissue-pattern of expression conservation among different species [85], considering that StLaurent et al. [38] used mouse lung tissues and we have used human kidney tumor tissues. Although lncRNAs are much less conserved than other functional ncRNAs such as miRNAs and snoRNAs [86], there is good evidence in the literature regarding the presence among the intronic lncRNAs of evolutionarily conserved regions spanning 400 nt or more [39,85,87]. Our recent work with pancreatic cancer has identified an enrichment of conserved regions within intronic and intergenic lncRNAs [46], and here we extend the identification of conserved regions to the intronic antisense lncRNAs expressed in RCC. Although some of the introns could contain regulatory sequences, or yet undiscovered coding exons overlapped by the intronic RNAs, thus accounting for part of the enrichment signal, the observed primary and secondary structure conservation suggests that the intronic lncRNAs are under the influence of evolutionary constraints.

In silico approaches have been successfully used to characterize sets of lncRNAs expressed in other tissues or cell lineages [9,28,29,46,69]. Here, we used them to obtain new data indicating that intronic lncRNAs should not be regarded simply as by-products of random transcription [38], but rather as a diverse and heterogeneous class of cellular transcripts that may comprise yet uncharacterized regulatory RNAs. The intronic lncRNAs identified here as expressed in RCC may have several mechanism of action, both positively and negatively regulating gene expression, and as a consequence, may constitute a promising starting point for further functional investigations.

## Material and methods

### Patient tissue material

Individual tissue samples were analyzed for gene expression with a 4 k-element array described below. The 29 tissue samples consisted of 18 primary renal tumors and 11 matched adjacent nontumor tissue from 18 patients who underwent radical nephrectomy for clear cell RCC at the Hospital of the Instituto Nacional de Cancer (INCa), Brazil. Ethical approval for the study was granted by INCa institutional review under the ID number 2701; all patients have signed an informed consent. Each sample was frozen and stored in liquid nitrogen immediately after surgery. A fraction of each sample was processed for histopathological diagnoses. A second independent histopathological diagnosis of each case was confirmed by a reference pathologist (GV) who belongs to the INCa staff. Histologically normal renal tissue fragments were collected from a distant portion of the surgically removed kidney. Clinical and anatomopathological patient data are detailed in Additional file 1: Table S1. The malignancy gene expression profile was identified with the 11 paired tumor and adjacent nontumor patient samples. Identification of the survival gene expression profile was performed with 16 tumor samples (nine tumors from the paired samples mentioned above for which the survival information was available, plus seven tumor samples for which only the survival information and not the paired nontumor tissue was available). Patient survival was recorded from the date of nephrectomy to the date the patient died or was last known alive (follow-up ranged from 60 to 66 months) and patients were identified as alive without disease (n = 8) or dead from cancer (n = 8). Kidney tissue samples expression was also measured with a 44 k-element oligo-array described below using 4 pools of nontumor (N) samples from 17 RCC, and 4 pools of the corresponding 17 paired tumor (T) samples. They comprise all 11 tumor-nontumor paired clear cell renal cell carcinoma (ccRCC) cases that were analyzed individually with the 4 k platform (Additional file 1: Table S1), plus other three ccRCC cases, two papillary RCC cases and one chromophobe RCC case. Clinical and anatomopathological data of these patients are detailed in Additional file 7: Table S5.

### Microarray platforms design

The custom 4 k-element microarray platform previously described by our group [45] is composed of 3355 unique cDNA probes from the Cancer EST Sequencing Project [88] spotted in duplicate (average length of 600 bp), plus positive and negative controls; 2371 probes interrogate cancer-related protein-coding genes compiled from the Entrez, OMIM and CGAP databases; an additional set of 984 probes was randomly sampled from cDNA clones whose sequences showed no similarity to protein-coding genes in GenBank, of which 722 are putative noncoding transcripts that map to intronic regions of known genes, 188 map to intergenic regions of the genome and 74 ESTs map to known RefSeq lincRNAs (a total of 262 putative lincRNAs). Probes were mapped and annotated according to the hg 19 assembly of the human Genome Reference Consortium (GRC) based on the RefSeq and UCSC datasets. The 4 k-array description is deposited in Gene Expression Omnibus (GEO) data repository under accession number GPL3985.

The custom 44 k-element oligoarray platform designed by our group and manufactured by Agilent Technologies was previously described [40]. Essentially, the array is comprised of strand-specific 60-mer oligonucleotide probes designed for both the plus or the minus genomic strands of 6,258 totally intronic noncoding (TIN) RNA *loci* and 4,267 partially intronic noncoding (PIN) RNA *loci* with evidence of transcription from dbEST [40], for a total of 21,050 strand-specific probes that interrogate both strands of 10,525 unique intronic *loci* within 6,361 unique protein-coding spliced human genes; the latter are represented by unique probes from the Agilent Whole Human Genome Oligo Microarray. Probes were mapped and re-annotated according to the hg 19 assembly of the human Genome Reference Consortium (GRC) based on the RefSeq and UCSC datasets. The 44 k-array description [40] and the re-annotation are deposited in the GEO repository under accession number GPL9193.

### Microarray experiments

Total RNA was isolated from frozen tissues with TRIzol reagent (Life Technologies) according to the manufacturer recommendations, followed by DNase I treatment for 20 min and purification with the RNeasy Mini kit (Qiagen). Purified total RNA was quantified in the NanoDrop ND-1000 spectrophotometer (Thermo Fisher Scientific), and checked for integrity with the 2100 Bioanalyzer (Agilent Technologies).

For the 4 k-element microarray assays, complementary RNA (cRNA) for each of the 29 samples (Additional file 1: Table S1) was obtained by linear amplification following the Wang protocol method [89]. Briefly, cDNA for each sample was synthesized from 3 μg total RNA using an oligo-dT primer incorporating a T7 RNA promoter and Superscript III Reverse Transcriptase (Invitrogen). Double-stranded cDNA was obtained using a template switch oligo primer with the Advantage cDNA Polymerase mix kit (Clontech). Subsequently, complementary RNA (cRNA) was generated *in vitro* with MegaScript T7 RNA Polymerase (Ambion). A second round of amplification was performed with 1 μg of cRNA obtained in the previous step, in the presence of amino-allyl-UTP (Ambion). Coupling of amino-allyl-cRNA with Cy5 reactive dye was performed (Amersham Pharmacia Biotech). Labeled cRNAs were purified using RNeasy Mini kit (Qiagen) and hybridized to a total of 29 microarray slides followed by washing and drying in an automated Hybridization Station (GE Healthcare) according to the manufacturer recommendations. Array images were acquired with a Generation III Array Scanner (GE Healthcare). Data were extracted from the scanned images with ArrayVision 6.0 software (GE Healthcare).

For the 44 k-element microarray assays, four pools of tumor (T1 to T4) or nontumor (N1 to N4) paired samples from 17 patients were assembled (three pools of 4 samples, one pool of five samples) as detailed in Additional file 7: Table S5 by mixing equal amounts of total RNA. Total RNA pool (300 ng) was used as template for the amplification of poly(A) RNA by the T7-RNA polymerase with the Low RNA Input Fluorescent Linear Amplification kit (Agilent Technologies), which generated Cy5- or Cy3-labeled cRNAs. A total of four array slides were hybridized with 750 ng each of Cy3- and Cy5-labeled cRNAs, in the following arrangement: Cy3 2T x 1N Cy5; Cy3 2N x 1T Cy5; Cy3 4T x 3N Cy5; Cy3 4N x 3T Cy5. Hybridization was performed with the Agilent *in situ* Hybridization kit-plus, as recommended by the manufacturer. The slides were washed and processed according to the Agilent Oligo Microarray Processing protocol and were scanned on a GenePix 4000B scanner (Molecular Devices). To extract intensity data from the scanned images we used the Agilent Feature Extraction software (Agilent Technologies). All the above microarray data are deposited at the GEO repository under the accession number GSE40914.

### Microarray data analyses

For the 4 k-element microarray, a gene was considered expressed if its probe intensity was higher than the local background intensity and above the threshold defined by the average intensity plus three standard deviations of a set of plant-derived negative control cDNA probes (GE Healthcare). Probes were excluded from further analyses when they were detected in less than 90% of the arrays in any of the two groups, i.e. nontumor or tumor for the malignancy analysis; or alive or dead from cancer for the survival analysis. The raw intensities were normalized by the quantile method [90].

For the 4 k-element microarray malignancy study, tumor/nontumor log_2_ ratios were calculated followed by a supervised one-class statistical analysis with the Significance Analysis of Microarrays (SAM) tool [91] with 1000 permutations to identify transcripts that were differentially expressed in eleven clear cell RCC and adjacent nontumor tissue. A sample leave-one-out cross-validation was performed [72,92]. Essentially, one sample was removed at a time, and each time a new set of significantly altered genes was determined with SAM using the remaining ten samples. This procedure was repeated for each of the matched tumor/adjacent nontumor tissue samples; a false discovery rate (FDR) cutoff ≤5% was used in all eleven leave-one-out datasets. This approach was used to minimize the contribution of each individual patient sample to the set of significantly altered genes [51]. The final gene profile is comprised of altered genes present in 100% of the leave-one-out datasets plus a 1.5-fold minimal change criterion. For the 4 k-element microarray survival study: a two-class unpaired Significance Analysis of Microarrays (SAM) analysis (FDR <10%) [91] combined with the Golub’s discrimination score analysis (p < 0.01) [93] was used for identifying transcripts expressed in clear cell RCC samples that were significantly correlated with the patient survival outcome. Only those genes found in common in both analyses were used to compose a profile of genes correlated to the outcome. The 16 patient samples were ordered in the heat-maps according to the correlation of their gene expression profiles to the average expression profile obtained from the 8 samples of patients who died from the disease within the 5-year follow-up after surgery.

For the 44 k-element oligoarray, the transcripts were considered expressed if the intensity of the spot was above the mean intensity plus 2 SD of the negative control spots in 3 out of 4 oligoarrays in one of the two groups (tumor or nontumor pools). For the intronic lncRNA transcripts, only the probe mapping to the genome in the antisense direction relative to the protein-coding mRNA in the *locus* was considered for further analyses.

### Real time RT-PCR

Reverse transcription was performed with 1 μg aliquots of DNase I-treated purified total RNA from the same paired samples that were used in the microarray experiments, oligo-dT primers and SuperScript III (Invitrogen) according to the manufacturer’s instructions. For the relative quantification of transcript levels, real-time PCR was performed using Power SYBR Green PCR Master Mix (Applied Biosystems) on an ABI PRISM 7500 machine (Applied Biosystems) and the following primers: *ncC11orf49*, GAGAAGCAGCGATGACACGAT (Forward), AGAGGAGCAACCCTCAGGAAA (Reverse); *HDAC5* exon 24/25, TGCAGCAAAAGCCCAACAT (Forward), AGACCAGCGGCGAACTTCT (Reverse); *ncHDAC5*, TATTCTGGAGTCGCCTGTGCTT (Forward), AACCACAGCCCTATTGGTATGC (Reverse); *ncRAB31*, CCCAGTGAGAGTGATATTTTGTTATGA (Forward), CCACACCTTCTTTCTGCCTGTT (Reverse); *ncSRPK1*, CAAGGGCTGAGTCCTTTTTCA (Forward), GCAGTGCCTTGCCCTTATTG (Reverse); *HPRT1*, TGACACTGGCAAAACAATGCA (Forward), GGTCCTTTTCACCAGCAAGCT (Reverse). The reactions were incubated at 95°C for 15 min, prior to 40 PCR cycles (15 sec at 95°C, 60 sec at 60°C). All reactions were performed in triplicates in a final volume of 20 μl containing 5 μl of diluted cDNA (1:3) and 800 nM of forward and reverse gene-specific primers. The gene expression levels of hypoxanthine phosphoribosyl transferase 1 (*HPRT1*) were used as a control to normalize the measurements. Transcript levels were expressed following the 2^-ΔΔCt^ method [94], where ΔΔCt = (ΔCt _candidate gene in tumor sample_ - ΔCt _candidate gene in nontumor sample_), with ΔCt = Ct candidate gene - Ct HPRT1.

### Orientation-specific reverse transcription

For the orientation-specific cDNA synthesis of *ncHDAC5*, *ncC11orf49*, *ncRAB31* and *ncSRPK1*, 1 μg of purified total RNA pretreated with DNase I (RNeasy Mini kit, Qiagen) was used for the reverse transcription reaction. A pool of RNA from 10 ccRCC samples or from 10 adjacent nontumor tissues samples was used as templates for the cDNA synthesis. In addition, purified DNaseI-treated total RNA from tumor kidney cell lineage 786-O or from nontumor cell lineage RC-124 was used as template. For each sample, two cDNA synthesis test reactions were performed, each with 1 μg of total RNA and 500 nM of an oligonucleotide primer complementary to the sequence of the lncRNA that would be transcribed from either the sense or the antisense strand within the corresponding *loci* of interest (see PCR primers above). SuperScript III Super Mix kit (Invitrogen) was used according manufacturer’s instructions. To avoid RNA self-annealing, pre-incubation of RNA and primer in annealing buffer was performed at 65°C for 10 min followed by the addition of reverse transcriptase in enzyme buffer pre-warmed at 57°C, and the reaction was incubated for 50 min at 57°C and denatured at 95°C for 10 min. To verify the absence of self-annealing or of genomic DNA contamination, a control reverse transcription reaction was performed in parallel without the addition of primers. These test and control samples were used for end-point PCR (40 cycles) with the pair of primers for the corresponding lncRNA, as described above.

### RACE-PCR

The Human Fetal Kidney Marathon-Ready cDNA library (Clontech) and the Marathon cDNA Amplification Kit (Clontech) were used to perform the 3′- and 5′ RACE-PCR, following the manufacturer instructions with the following primers: HDAC5_F_GSP_RACE: AGGAGCCCTGCAGAGAGCACATGG; HDAC5-F_Nested_RACE: AAGGGGAATCTCCCACCAGCCTGTC; HDAC5-R_GSP_RACE: GGGGTGCTGCATGTCACCCAGTC; HDAC5-R_Nested_RACE: TGGAGTCGCCTGTGCTTCCTGTTTG.

### RNA stability assay

786-O cells were maintained at exponential growth in Dulbecco’s modified Eagle’s medium (DMEM) containing 10% calf serum, penicillin, and streptomycin. Actinomycin D was dissolved in DMSO and added to cells at 5 μg/ml. Cells were collected at time 0 h (before actinomycin D treatment), 30 min, 1 h, 2 h and 4 h. Total RNA was extracted and DNAse I treated with RNeasy Mini kit (Qiagen). cDNA was obtained with SuperScript III Super Mix kit (Invitrogen) according to manufacturer’s instructions. These cDNAs were used for real-time PCR with the pair of primers for *ncHDAC5* as described above. As a control of the assay, the half-life for the *C-MYC* transcript was checked, and the expected value of ~30 min was obtained.

### In silico analyses

To search for protein coding potential of the expressed antisense lncRNAs in renal cancer we used the Coding Potential Calculator (CPC) software [64] with default parameters. To search for RefSeq annotation we mapped the genomic coordinates of our 4303 antisense lncRNA set to the RefSeq UCSC database (http://genome.ucsc.edu/). To identify possible precursors of small RNAs among our set of lncRNAs we cross-referenced the genomic coordinates of our 4303 antisense lncRNA set to snoRNA [62] and microRNA [63] databases, using the sno/miRNA (wgRNA) UCSC track (http://genome.ucsc.edu/). For the gene expression meta-analysis we used the Oncomine™ Gene Browser software tool (http://www.oncomine.org).

We investigated the co-expression pattern of intronic antisense lncRNAs and mRNAs, both in *cis* (lncRNA and mRNA from the same *locus* expressed in a given tissue) and in *trans* (each lncRNA and all mRNAs expressed in a tissue). First, we created a list of all the antisense lncRNAs expressed in RCC, and identified those that were also expressed in other three tissues, namely nontumor kidney, normal liver and prostate tumor human tissues (GEO: GSE5452), using the normalized microarray expression data previously obtained by our group [40].

For the *in cis* correlation analysis, we used the data from RCC and the other three tissues and calculated the Spearman correlation (ρ) using the R software environment (http://www.r-project.org), with a cutoff of -0.5 > ρ >0.5 (p <0.05). We used GraphPad Prism software (GraphPad Softwares, La Jolla, California, USA) to obtain the histogram of Spearman correlation distribution *in cis.* With the Bingo software [95], we identified enriched Gene Ontology terms (p <0.05) considering the set of protein-coding genes co-expressed *in cis* (-0.5 > ρ >0.5; p <0.05), the GO_Biological_Process ontology file and the whole human genome annotation default Bingo 2.44 version datasets.

For the *trans* correlation analysis, we only considered the top 20% most abundant antisense lncRNAs in RCC. We constructed a matrix of correlation (using a R script) of 693 antisense lncRNAs versus 5438 mRNAs expressed in RCC and in the other three tissues described above. Next, we selected the lncRNAs most correlated *in trans* (cutoff -0.7 ≥ ρ ≥0.7, p <0.05) and used the Genomica software (http://genomica.weizmann.ac.il) [96] to identify among the correlated mRNAs the sets of genes (modules) that were significantly enriched (p <0.05 with Bonferroni correction) for a specific Gene Ontology term from the three ontologies, namely Biological Processes, Molecular Function and Cellular Component.

For transcription regulatory elements and conservation analyses, we used the BEDTools software package [97] to compare the genomic coordinates (hg19 GRCh37) of our antisense lncRNAs dataset with the genomic coordinates of the following datasets available at UCSC Genome Browser: RIKEN CAGE tags [98] from PolyA + RNA-derived libraries from 35 cell lines released by the ENCODE project [65]; predicted CpGs islands [66]; HMM active promoter prediction [59]; RNA Polymerase II binding site from the transcription factor ChIP-seq uniform peaks ENCODE track for 32 human cell lines [65]; ChIP-seq data of H3K27ac and H3K4me1 DNA binding sites from seven different human cell lines [59]; H3K4me3, H3K36me3 and H3K27me3 DNA binding sites from human renal epithelial cells [60]; RNA-seq data of PolyA + RNA-derived libraries from 9 tissues [68]; RNA-seq data of strand-oriented RNA-derived libraries from 7 cell lines [69].

To test for the statistical significance of the overlap distribution (see below), we created 10 control datasets of randomly selected sequences from the entire human genome matching our set of expressed antisense lncRNA sequences in number and length. Regulatory elements mapping up to 1 kb upstream from TSS and 5′UTRs of RefSeq known transcripts were removed to avoid the contribution of signals at the start sites of known genes to the enrichment of regulatory elements at the start sites of lncRNAs mapping nearby. As a pre-processing step of the CAGE tag data analysis, only CAGE tags that presented RPKM (reads per kilobase per million) ≥ 1 were considered for further analysis [69]. We computed the distance of the closest CAGE tags, CpG islands and HMM predicted active promoter, RNA Pol II, H3K27ac, H3K4me1, H3K4me3 and H3K27me3 marks to the predicted TSSs of our set of 4303 expressed antisense lncRNAs, 11102 isoforms from 5632 expressed protein-coding mRNAs, and control sets of 4303 random sequences. Regulatory elements distant up to 10 kb of the sequence initiation were considered. For the H3K36me3 mark, the number of overlapping elements was recorded. Those records were used to create a distribution of overlaps for all lncRNAs, binned into 1-kb intervals. The Kolmogorov-Smirnov (KS) test statistics was used to compare continuous probability distributions of abundance of each relevant genomic mark with those calculated for each of the 10 control random sets (p-values < 0.05 threshold).

To evaluate the tissue specificity of antisense lncRNAs and protein-coding mRNAs expressed in renal cancer a meta-analysis including Burge’s RNA-seq data from nine human tissues [68] and strand-oriented Caltech RNA-seq libraries from seven human cell lineages [69] was performed. For the Burge RNA-seq data, we mapped the transcripts to the hg19 reference genome (hg19 GRCh37) using TopHat [99] and assembled the transcripts using cufflinks [100]. RefSeq mRNA (October 2012, UCSC) plus intronic antisense lncRNAs comprised in the 44 k array were used as the reference transcripts. To determined tissue specificity we used an approach similar to Marques and Ponting [101]; thus we calculated the fraction of expression in each tissue (F.E.T.) as being the FPKM observed in a specific tissue divided by the sum of FPKMs in all tissues. To address statistical significance, we performed Fischer’s exact test comparing the rates of F.E.T. ≥ 0.5 between the lncRNAs and the protein-coding mRNAs (p < 0.001). The genomic coordinates of antisense lncRNAs (or mRNAs) expressed in RCC were overlapped with the coordinates of the Caltech RNA-seq data to determine if the transcript was identified in each of the strand-oriented RNA-seq libraries.

Conservation of expression pattern analyses were performed by mapping the sequence coordinates of antisense lncRNAs expressed in RCC to the coordinates of transcripts expressed in humans and in 15 other vertebrate species, as compiled in the TransMap cross-species syntenically mapped cDNA alignments [70], and recording the hits in each species. To determine the statistical significance of expression pattern conservation, we compared the number of hits against the 15 species obtained for the lncRNA sequences with the number of hits against the 15 species obtained for 10 random sets of sequences with identical length as those of the lncRNAs. Fischer’s exact test (p < 0.05 threshold) was used.

Conservation of expression pattern of intronic antisense lncRNAs between RCC and intronic antisense lncRNAs expressed in mouse [38] was identified by transposing the mouse genomic coordinates to the human genome using the liftOver tool (http://genome.ucsc.edu/cgi-bin/hgLiftOver), and the overlap between these transcripts and the set of intronic antisense lncRNAs expressed in RCC was determined using intersectBed from the BEDtools package [97]. The same analysis was done with the coordinates from a random set of 4303 sequences extracted from the subset of probes with no evidence of expression in RCC among the entire set of 10,525 intronic antisense lncRNAs probed in the 44 k array. Fisher test was used to determine statistical significance (p < 0.05 threshold).

The analysis of DNA sequence conservation was performed by cross-referencing the human genome coordinates of antisense lncRNAs expressed in RCC with the coordinates of PhastCons DNA conserved elements from vertebrates, from placental mammalians and from primates [61], and counting the number of overlaps. To determine the statistical significance, the coordinates from the 10 random sets described above were analyzed in the same way against PhastCons dataset. Fischer’s exact test (p < 0.05 threshold) was used.

RNAz tool [71] was used to predict structurally conserved and thermodynamically stable RNA secondary structures. Only predicted structures with P (Probability) > 0.5 were considered as containing conserved secondary structures [71].

## Competing interests

The authors declared they have no competing interests.

## Authors’ contributions

AAF designed the study, carried out microarray and RT-PCR experiments, performed in silico analyses and drafted the manuscript; ACT participated in microarray experiments and performed in silico analyses; SAVA participated in RT-PCR and performed *ncHDAC5* characterization experiments; VMC performed in silico analyses; EG obtained clinical patient information; GV carried out histological tissue classification; FSC helped to conceive the study and obtained patient agreement and tissue samples for the study; EMR participated in the design and coordination of the study; SVA conceived and coordinated the study, and drafted the manuscript with input from all authors. All authors read and approved the final manuscript.

## Supplementary Material

Additional file 1: Table S1 Clinical and pathological data for the 18 clear cell RCC patients analyzed with the 4 k-element cDNA microarrays. Click here for file

Additional file 2: Figure S1 Protein-coding gene expression signature of ccRCC. Heat map of 217 differentially expressed protein-coding genes (rows) identified in 11 ccRCC patients (columns) (FDR <5%; 1.5-fold change). Patient ID numbers are indicated at the bottom. Blue indicates lower expression, and red, higher expression in tumor (T) tissue in relation to adjacent nontumor (N) tissue. Click here for file

Additional file 3: Table S2 List of 217 protein-coding genes differentially expressed in ccRCC in the microarray analysis. Click here for file

Additional file 4: Figure S2 Expression signature of intronic lncRNAs correlated to patient survival in ccRCC. (A) A set of 26 intronic lncRNAs (rows) identified as differentially expressed (FDR ≤5%; p <0.01) between two ccRCC patient groups with distinct outcomes, namely alive and disease-free or dead from cancer within a 5-year follow-up period after surgery. Patient samples (columns) are ordered by their correlation relative to the mean expression profile of the group of patients that died from cancer. The color code shows higher (red) or lower (blue) expression relative to the mean expression of that lncRNA in all patients. (B) Clinical and pathological features: PS, Patient Status (white = alive disease-free; black = cancer death); T, primary tumor classification (white = 1a/1b; black = 2/3a/3b/3c); N, regional lymph node positive for metastasis (white = no; black = yes); M, presence of metastasis at surgery (white = no; black = yes); Necr, presence of necrosis (white = no; black = yes); Sz, primary tumor size (white ≤ 7 cm; black > 7 cm); FG, Fuhrman’s nuclear grade (white = II; black = III/IV); Age, age at surgery (white ≤ 60-year-old; black > 60-year-old); Gend, Gender (white = female; black = male). (C) Correlation coefficient (r) of each sample in relation to the average expression profile of all samples from patients who died from the disease. Patient samples were ordered according to this correlation. Click here for file

Additional file 5: Table S3 List of 26 intronic lncRNAs significantly correlated to RCC patient’s survival outcome identified through a cancer-related death analysis of the microarray data. Click here for file

Additional file 6: Table S4 *ncHDAC5* lncRNA half-life measured in a human renal cell line following transcriptional inhibition with actinomycin D and *ncHDAC5* lncRNA conserved secondary structure predictions calculated with the RNAz tool. Click here for file

Additional file 7: Table S5 Clinical and pathological data of the 17 RCC patients analyzed with the 44 k-element oligoarrays. Click here for file

Additional file 8: Table S6 List of 4303 intronic antisense lncRNAs expressed in RCC. Information is provided for the evolutionary conservation and for the *cis*-correlated analysis of expression in four tissues. Click here for file

Additional file 9: Figure S3 All significantly enriched GO terms identified for the set of protein-coding genes expressed in RCC and also in other three human tissues (normal liver, prostate tumor and kidney nontumor) and *cis* correlated (-0.5 > ρ >0.5; p < 0.05) with antisense lncRNAs from the same *loci*. GO enriched terms are organized in Subgroup I: biological regulation; Subgroup II: cellular process; Subgroup III: developmental process. Subgroup IV: GO enriched terms for protein-coding genes only showing positive *cis* correlation with the antisense lncRNAs. Subgroup V: GO enriched terms for protein-coding genes only showing negative *cis* correlation with the antisense lncRNAs. Color scale indicates increasingly higher statistical significance of enriched GO terms: Yellow, p =0.05; Dark orange, p <0.0001. Click here for file

Additional file 10: Table S7 GO enriched terms from *cis* or *trans* correlation analyses. (A) GO enriched terms for all protein-coding genes with significant *cis* correlation with the lncRNA from the same *locus*. (B, C) GO enriched terms for protein-coding genes with significant *cis* correlation with the lncRNA from the same *locus*, for those only with (B) positive correlation or (C) negative correlation among lncRNAs and protein-coding genes. (D) Average *trans* correlation value for the protein-coding gene set expressed in RCC and other three tissues, belonging to that GO-enriched term, and having significant *trans* correlation with a lncRNA. (E) Correlation values for all lncRNAs with significant *trans* correlation with all the protein-coding genes expressed in RCC plus other three tissues. (F) Protein-coding genes expressed in RCC plus other three tissues, belonging to that GO-enriched term in the *trans* correlation analysis. Click here for file

Additional file 11: Figure S4 Heat map of *trans*-correlated expression among the 20% most abundant antisense lncRNAs (n = 693) expressed in RCC and other three tissues and the 5293 protein-coding mRNAs expressed from different *loci*. A yellow entry indicates a Spearman correlation ρ ≥0.7; a blue entry indicates a Spearman correlation ρ ≤ -0.7; a black entry indicates all other correlation values. A total of 693 antisense lncRNAs and 5293 mRNAs expressed in the four tissues were considered in the *trans*-correlation analysis. Click here for file

Additional file 12: Figure S5 Tissue expression pattern of protein-coding genes. (A) Heat map representing abundance of 5632 RCC-expressed protein-coding genes (columns) across other nine human tissues (rows) from public RNA-seq libraries [68]. Color intensity represents fractional density expression of each lncRNA across all tissues (see Material and methods for details). (B) Heat map indicating presence (red) or absence (white) of 5298 RCC-expressed protein-coding genes (columns) across seven human cell lineages (rows) from public strand-oriented RNA-Seq libraries [69]. Expression data was hierarchically clustered. Click here for file
