# Supplementary figures and images for: Expression analysis and in silico characterization of intronic long noncoding RNAs in renal cell carcinoma: emerging functional associations
Source: Mol Cancer. 2013 Nov 15;12:140. doi: 10.1186/1476-4598-12-140 (PMC3834536; doi:10.1186/1476-4598-12-140)

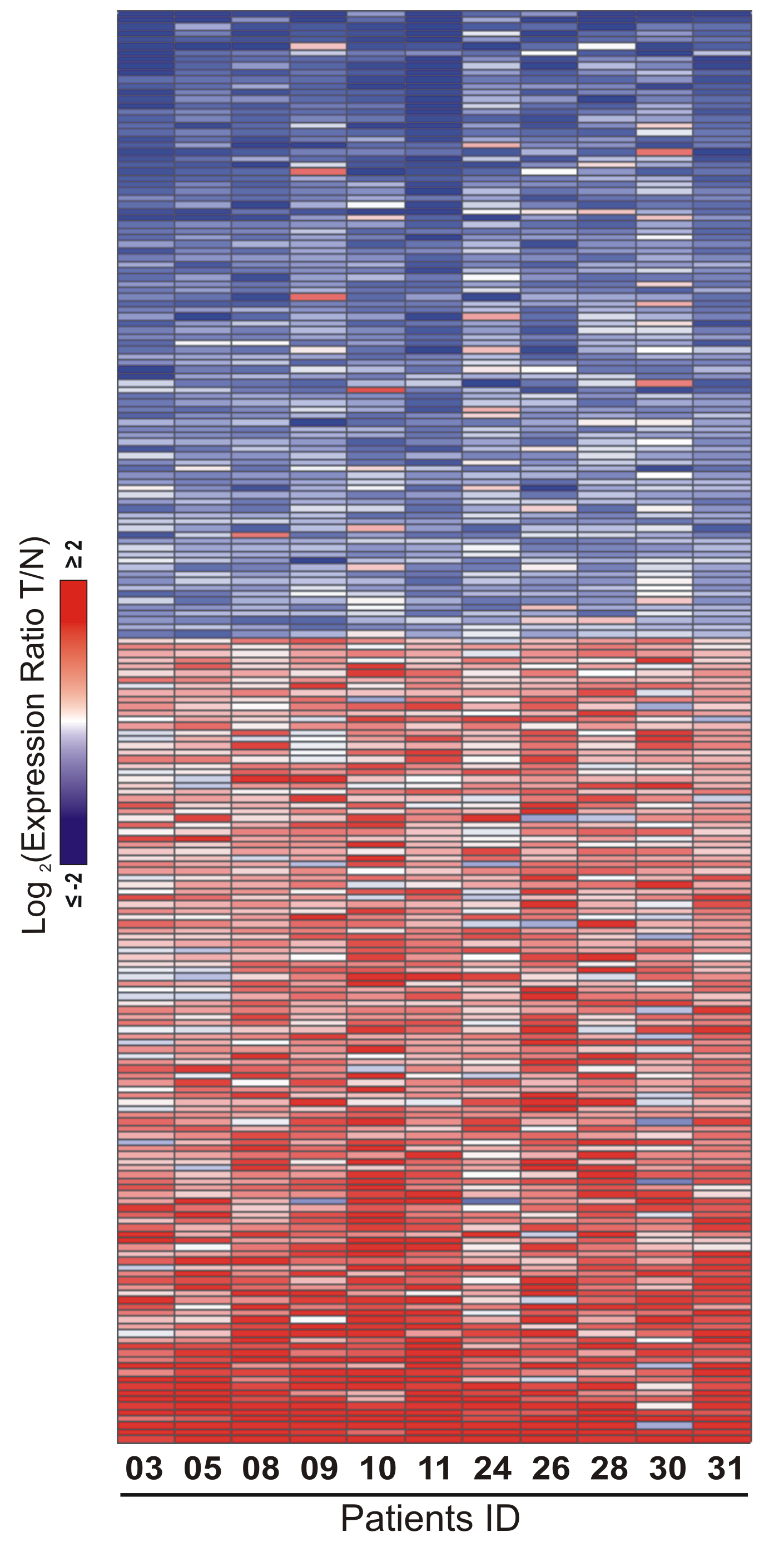

Supplement: Additional file 2: Figure S1 — Protein-coding gene expression signature of ccRCC. Heat map of 217 differentially expressed protein-coding genes (rows) identified in 11 ccRCC patients (columns) (FDR <5%; 1.5-fold change). Patient ID numbers are indicated at the bottom. Blue indicates lower expression, and red, higher expression in tumor (T) tissue in relation to adjacent nontumor (N) tissue. [file 1476-4598-12-140-S2.tiff]

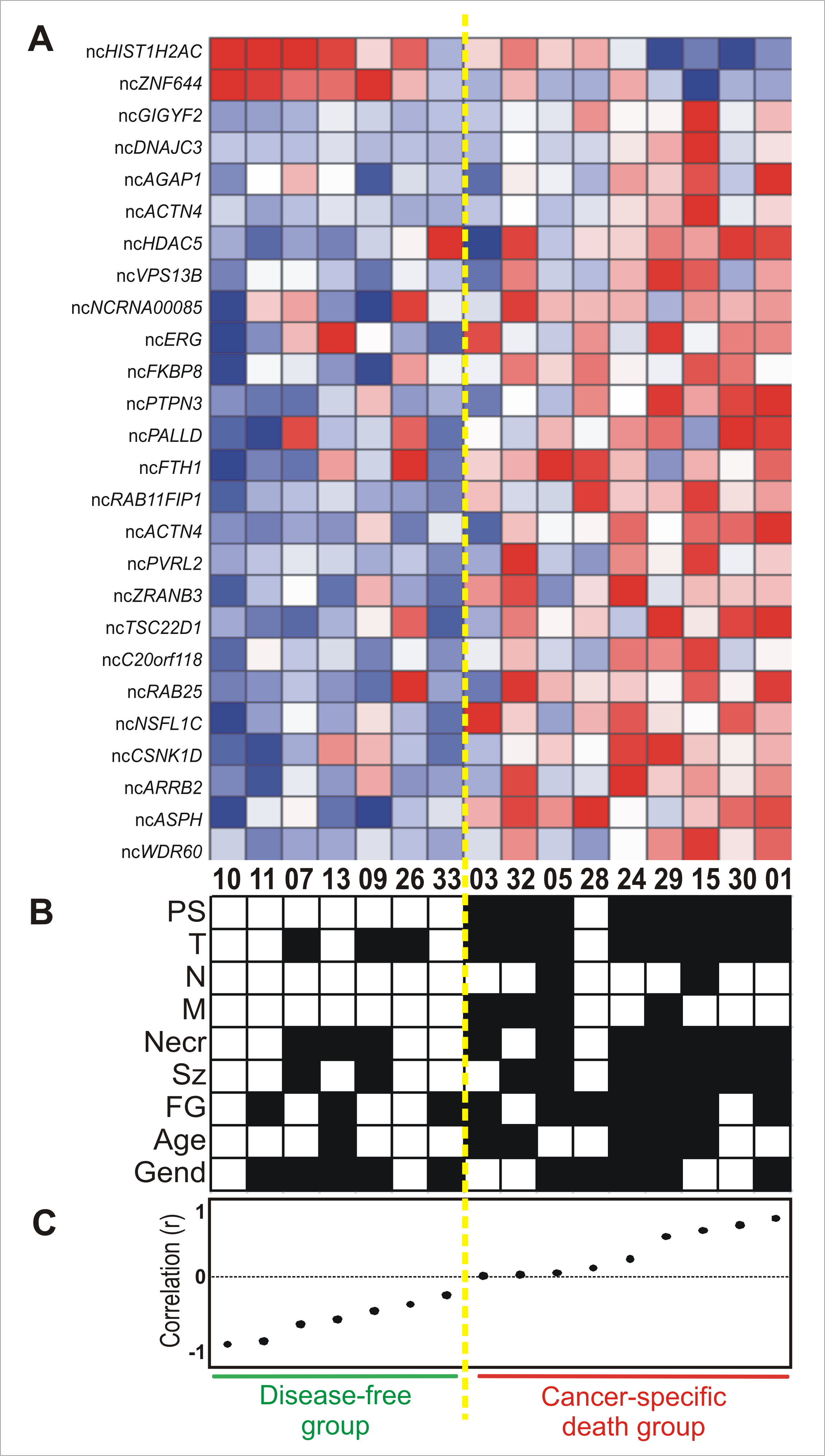

Supplement: Additional file 4: Figure S2 — Expression signature of intronic lncRNAs correlated to patient survival in ccRCC. (A) A set of 26 intronic lncRNAs (rows) identified as differentially expressed (FDR ≤5%; p <0.01) between two ccRCC patient groups with distinct outcomes, namely alive and disease-free or dead from cancer within a 5-year follow-up period after surgery. Patient samples (columns) are ordered by their correlation relative to the mean expression profile of the group of patients that died from cancer. The color code shows higher (red) or lower (blue) expression relative to the mean expression of that lncRNA in all patients. (B) Clinical and pathological features: PS, Patient Status (white = alive disease-free; black = cancer death); T, primary tumor classification (white = 1a/1b; black = 2/3a/3b/3c); N, regional lymph node positive for metastasis (white = no; black = yes); M, presence of metastasis at surgery (white = no; black = yes); Necr, presence of necrosis (white = no; black = yes); Sz, primary tumor size (white ≤ 7 cm; black > 7 cm); FG, Fuhrman’s nuclear grade (white = II; black = III/IV); Age, age at surgery (white ≤ 60-year-old; black > 60-year-old); Gend, Gender (white = female; black = male). (C) Correlation coefficient (r) of each sample in relation to the average expression profile of all samples from patients who died from the disease. Patient samples were ordered according to this correlation. [file 1476-4598-12-140-S4.tiff]

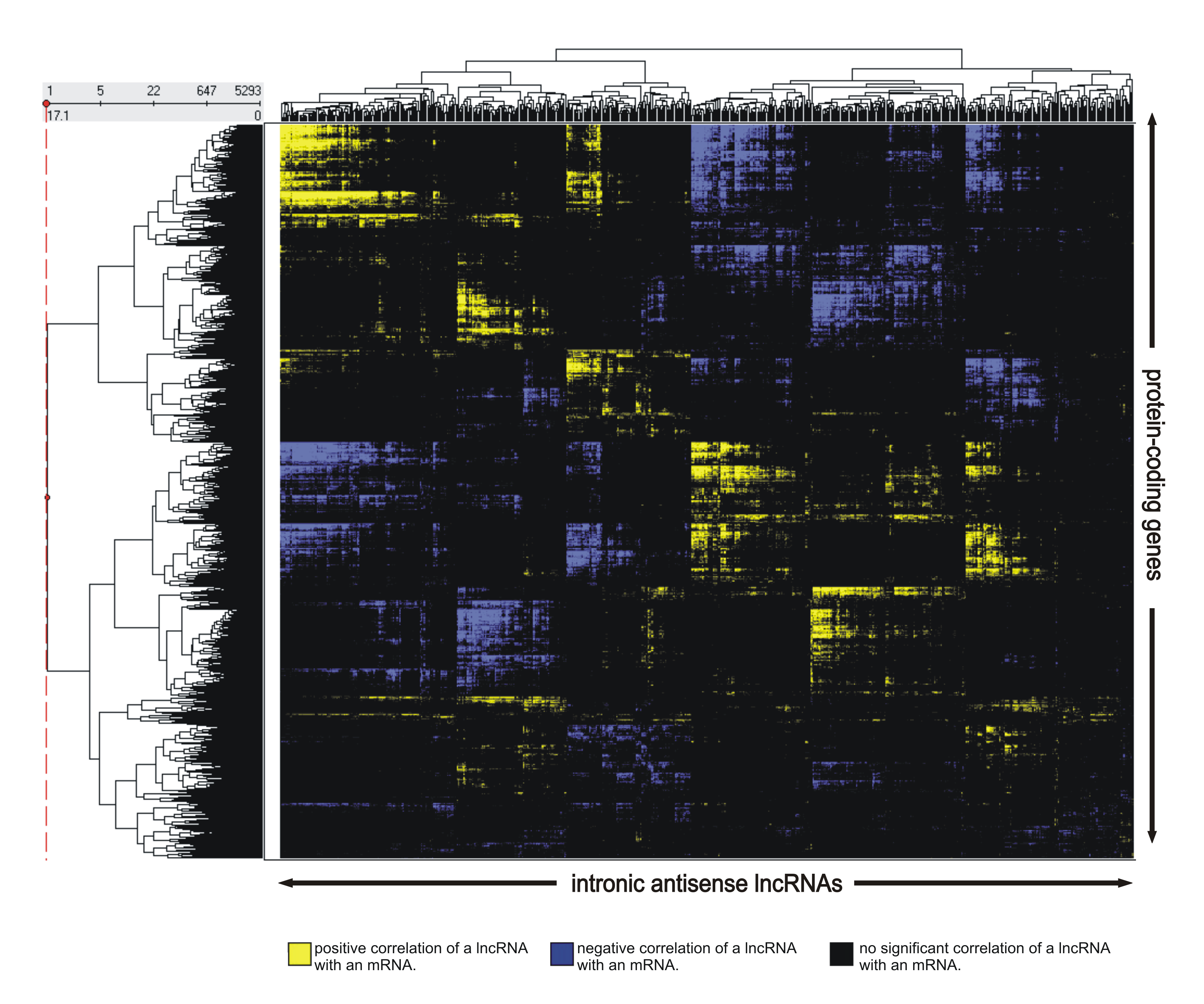

Supplement: Additional file 11: Figure S4 — Heat map of trans-correlated expression among the 20% most abundant antisense lncRNAs (n = 693) expressed in RCC and other three tissues and the 5293 protein-coding mRNAs expressed from different loci. A yellow entry indicates a Spearman correlation ρ ≥0.7; a blue entry indicates a Spearman correlation ρ ≤ -0.7; a black entry indicates all other correlation values. A total of 693 antisense lncRNAs and 5293 mRNAs expressed in the four tissues were considered in the trans-correlation analysis. [file 1476-4598-12-140-S11.tiff]

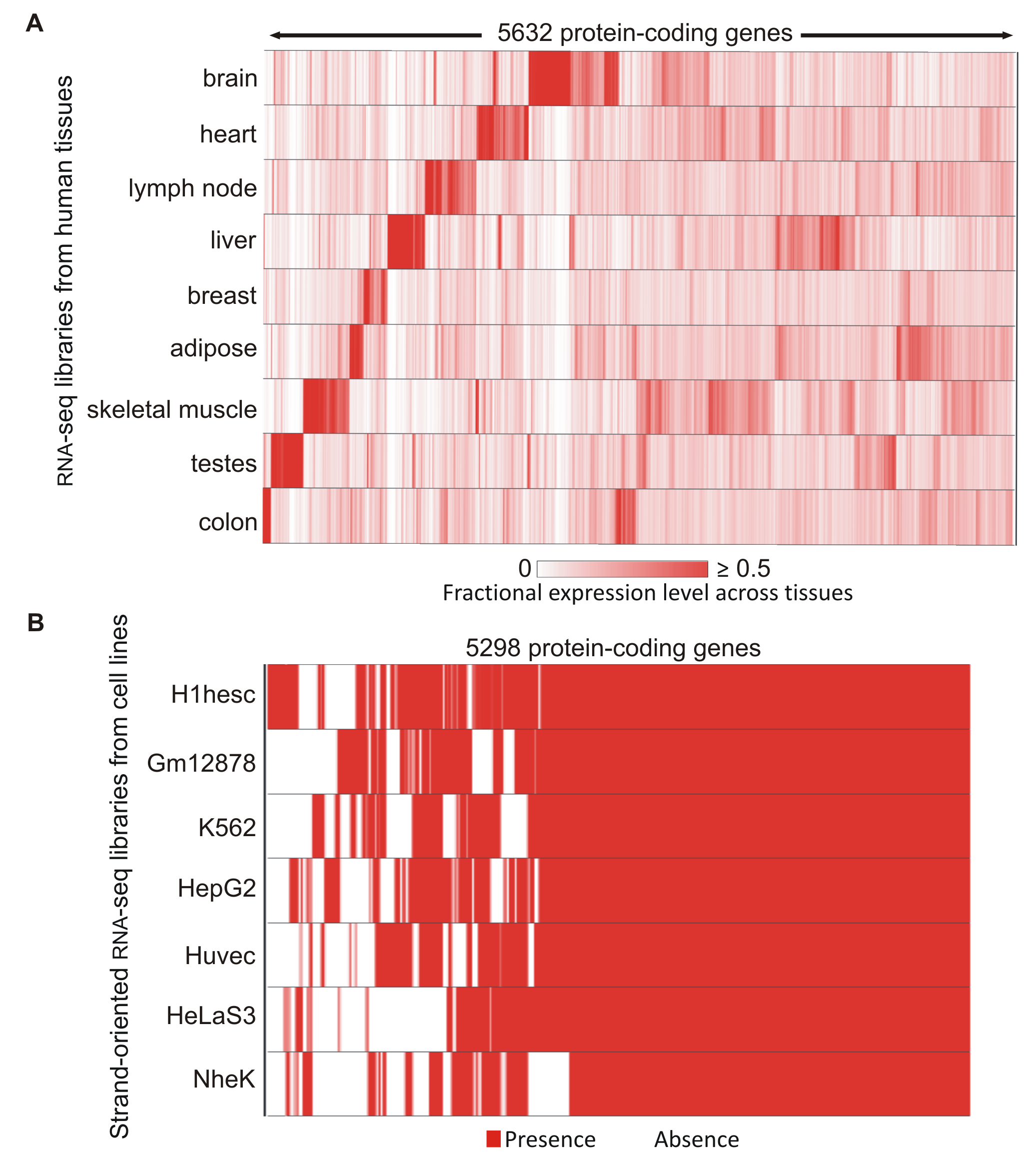

Supplement: Additional file 12: Figure S5 — Tissue expression pattern of protein-coding genes. (A) Heat map representing abundance of 5632 RCC-expressed protein-coding genes (columns) across other nine human tissues (rows) from public RNA-seq libraries [68]. Color intensity represents fractional density expression of each lncRNA across all tissues (see Material and methods for details). (B) Heat map indicating presence (red) or absence (white) of 5298 RCC-expressed protein-coding genes (columns) across seven human cell lineages (rows) from public strand-oriented RNA-Seq libraries [69]. Expression data was hierarchically clustered. [file 1476-4598-12-140-S12.tiff]
