# Supplementary material for: Expression analysis and in silico characterization of intronic long noncoding RNAs in renal cell carcinoma: emerging functional associations
Source: Mol Cancer. 2013 Nov 15;12:140. doi: 10.1186/1476-4598-12-140 (PMC3834536; doi:10.1186/1476-4598-12-140)

Subgroup I: All *cis* correlations - GO enrichment analysis - Biological regulation

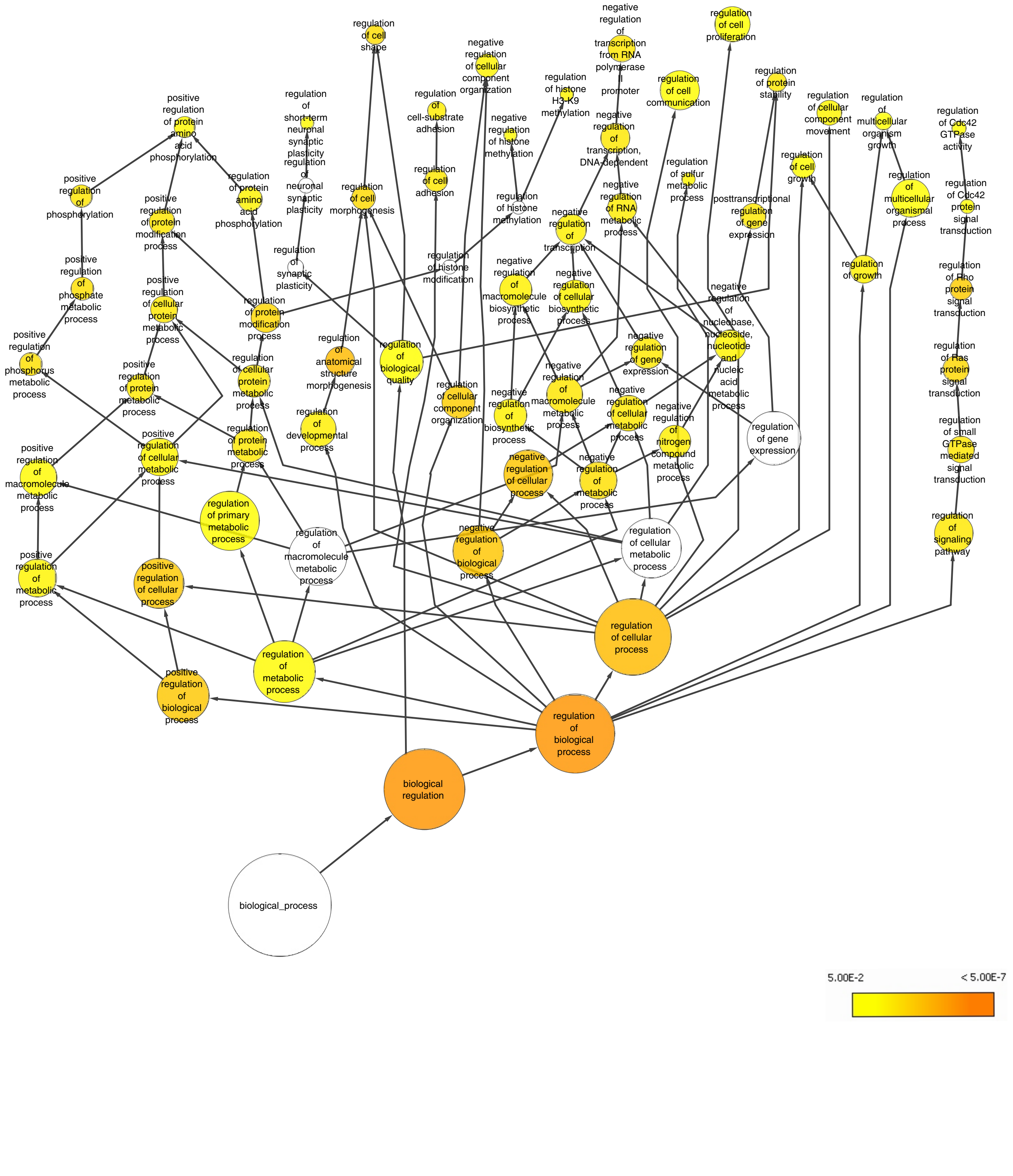

Subgroup II: All *cis* correlations - GO enrichment analysis - Cellular process

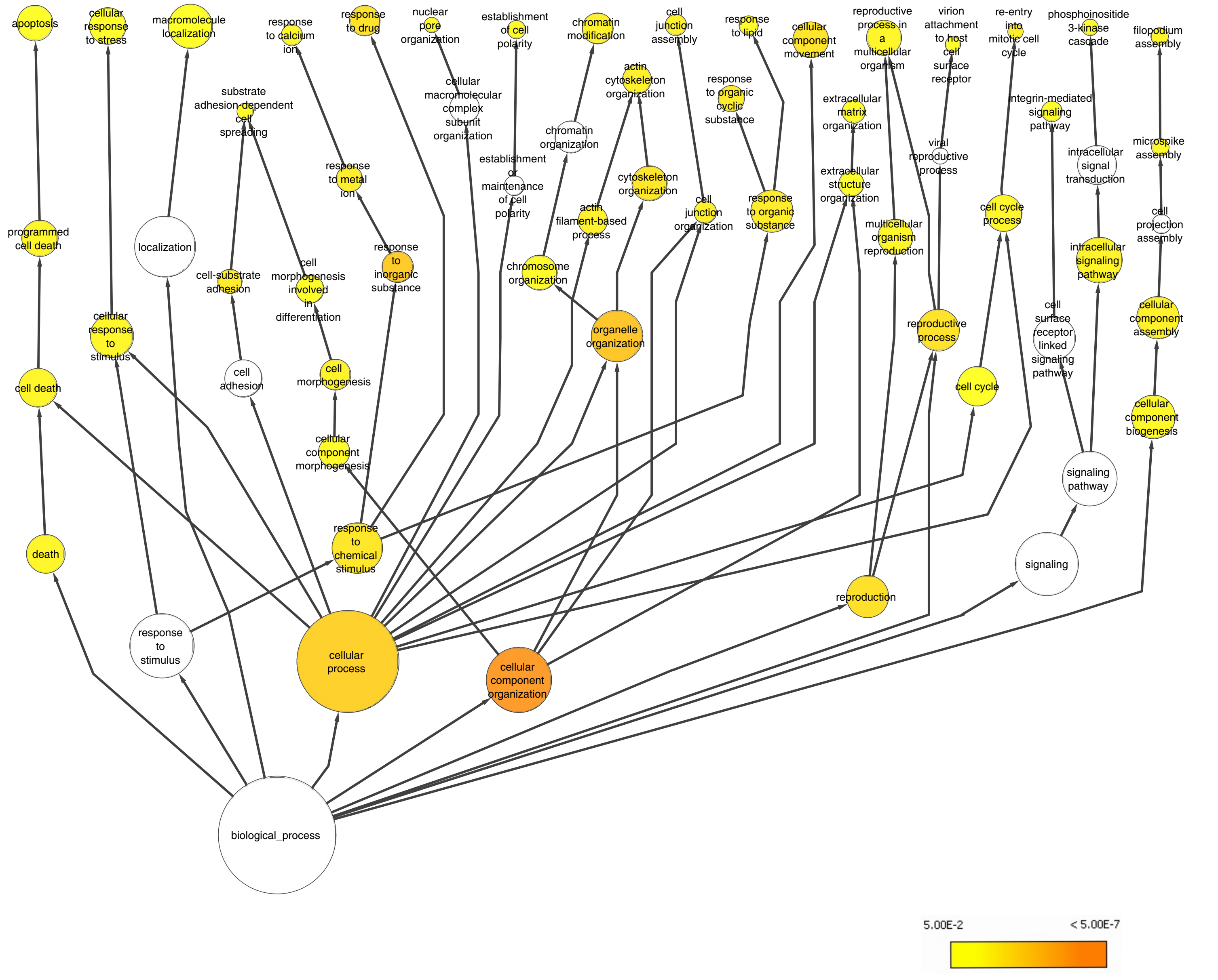

Subgroup III: All *cis* correlations - GO enrichment analysis - Developmental process

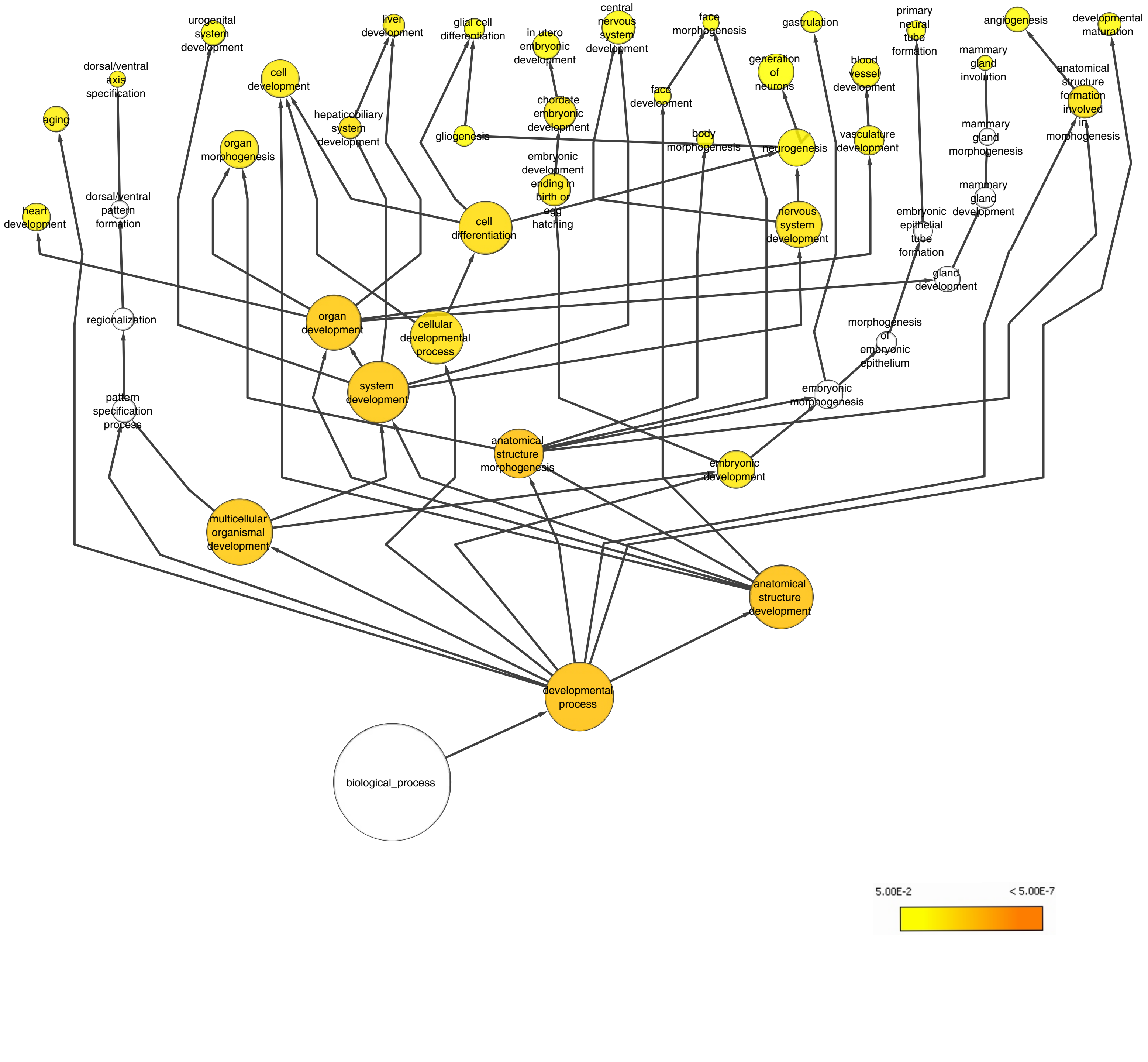

Subgroup IV: Positive *cis* correlations - GO enrichment analysis - Cellular and biological processes

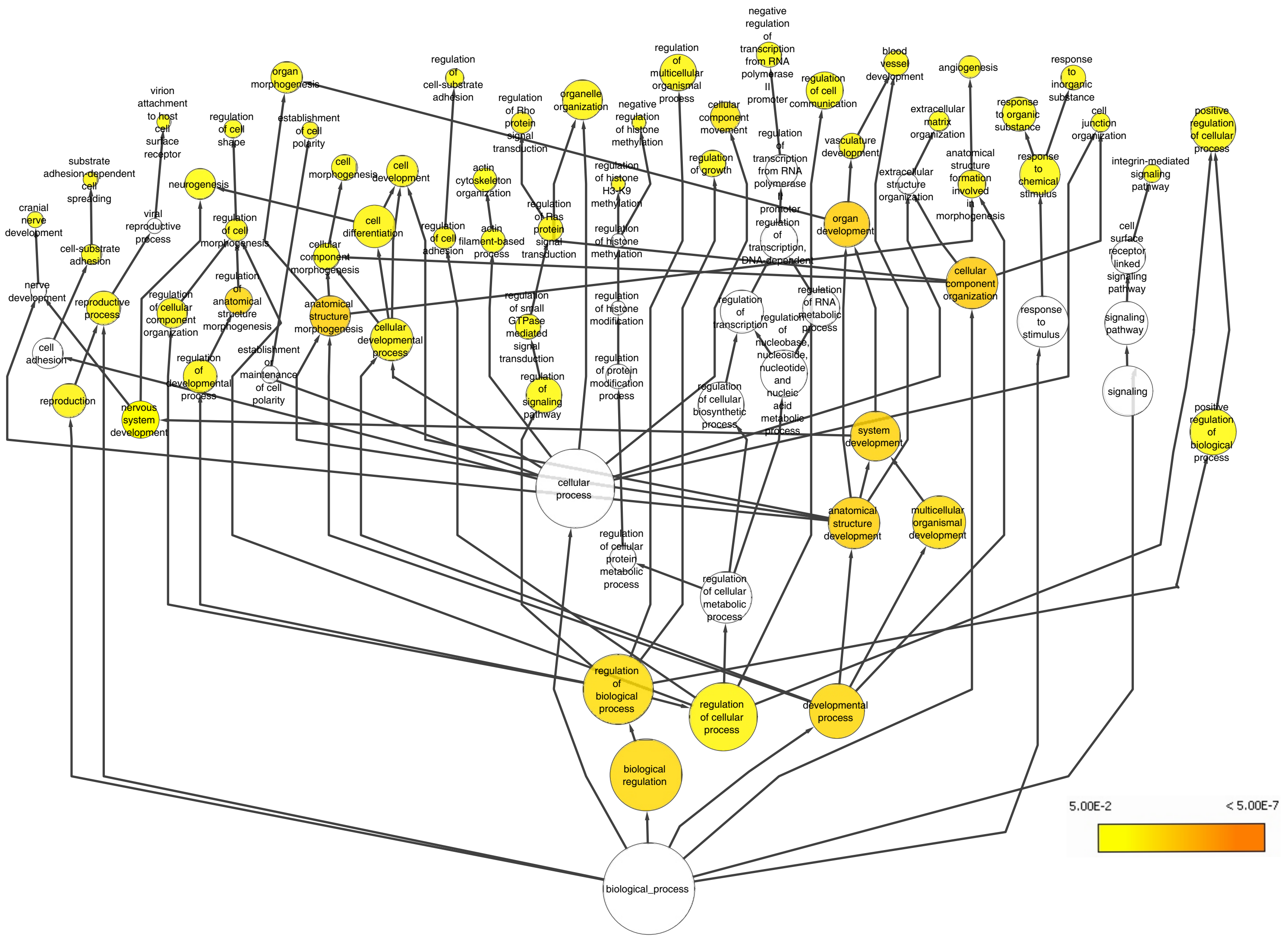

Subgroup V: Negative *cis* correlations - GO enrichment analysis - Metabolic process

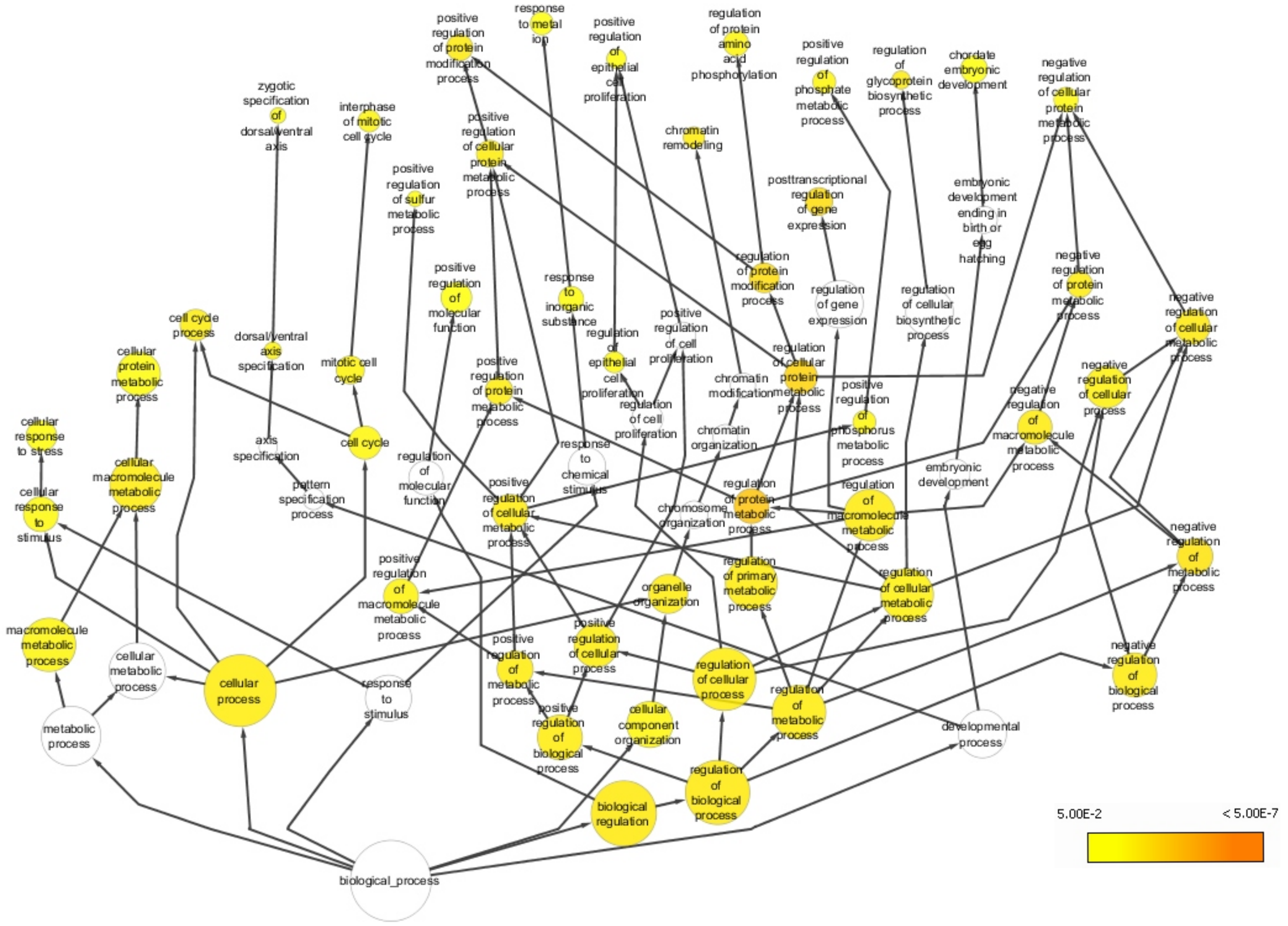

Supplement: Additional file 9: Figure S3 — All significantly enriched GO terms identified for the set of protein-coding genes expressed in RCC and also in other three human tissues (normal liver, prostate tumor and kidney nontumor) and cis correlated (-0.5 > ρ >0.5; p < 0.05) with antisense lncRNAs from the same loci. GO enriched terms are organized in Subgroup I: biological regulation; Subgroup II: cellular process; Subgroup III: developmental process. Subgroup IV: GO enriched terms for protein-coding genes only showing positive cis correlation with the antisense lncRNAs. Subgroup V: GO enriched terms for protein-coding genes only showing negative cis correlation with the antisense lncRNAs. Color scale indicates increasingly higher statistical significance of enriched GO terms: Yellow, p =0.05; Dark orange, p <0.0001. [file 1476-4598-12-140-S9.pdf]
